# *Neisseria gonorrhoeae* Limits *Chlamydia trachomatis* Inclusion Development and Infectivity in a Novel *In Vitro* Co-Infection Model

**DOI:** 10.3389/fcimb.2022.911818

**Published:** 2022-07-07

**Authors:** Delia Onorini, Nicole Borel, Robert V. Schoborg, Cory Ann Leonard

**Affiliations:** ^1^ Institute of Veterinary Pathology, Vetsuisse Faculty, University of Zurich, Zurich, Switzerland; ^2^ Department of Medical Education, Center for Infectious Disease, Inflammation and Immunity, Quillen College of Medicine, East Tennessee State University, Johnson City, TN, United States

**Keywords:** *Chlamydia trachomatis*, *Neisseria gonorrhoeae*, co-infection, *in vitro* model, inclusion development, infectivity

## Abstract

*Chlamydia trachomatis* (Ct) and *Neisseria gonorrhoeae* (Ng) are the most common bacterial sexually transmitted infections (STIs) worldwide. The primary site of infection for both bacteria is the epithelium of the endocervix in women and the urethra in men; both can also infect the rectum, pharynx and conjunctiva. Ct/Ng co-infections are more common than expected by chance, suggesting Ct/Ng interactions increase susceptibility and/or transmissibility. To date, studies have largely focused on each pathogen individually and models exploring co-infection are limited. We aimed to determine if Ng co-infection influences chlamydial infection and development and we hypothesized that Ng-infected cells are more susceptible to chlamydial infection than uninfected cells. To address this hypothesis, we established an *in vitro* model of Ct/Ng co-infection in cultured human cervical epithelial cells. Our data show that Ng co-infection elicits an anti-chlamydial effect by reducing chlamydial infection, inclusion size, and subsequent infectivity. Notably, the anti-chlamydial effect is dependent on Ng viability but not extracellular nutrient depletion or pH modulation. Though this finding is not consistent with our hypothesis, it provides evidence that interaction of these bacteria *in vitro* influences chlamydial infection and development. This Ct/Ng co-infection model, established in an epithelial cell line, will facilitate further exploration into the pathogenic interplay between Ct and Ng.

## Introduction

*Chlamydia trachomatis* (Ct) and *Neisseria gonorrhoeae* (Ng) are the most common bacterial sexually transmitted infections (STIs) worldwide, with an estimated 127 million and 87 million new cases of chlamydia and gonorrhea, respectively, among adults in 2016, as reported by the World Health Organization (WHO) (WHO; Rowley et al., 2019). For both bacteria, the primary infection site is the epithelium of the endocervix in women and the urethra in men. Additional infection sites include the rectum, pharynx, and conjunctiva in women and men (Holmes et al., 2008; Leonard et al., 2019). Associated syndromes for both STIs are typically cervicitis in women and urethritis in men, however, asymptomatic infections are common and may remain undetected. Though effective therapies currently exist for both STIs, antibiotic resistance for Ng is widespread, increasing rapidly with each successive treatment regimen (Jerse et al., 2015). As vaccines are unavailable, control of these infections remains a major global public health concern.

Ct is an obligate intracellular bacterium with a biphasic developmental cycle in which it alternates between two morphological forms: the elementary body (EB) and the reticulate body (RB). The EBs are small, metabolically less active, infectious, and responsible for attachment and invasion of susceptible cells. Upon infection of host cells, EBs are internalized in membrane-bound vacuoles referred to as “inclusions”, in which EBs differentiate into RBs. The RBs are large, metabolically active, and non-infectious forms which replicate and undergo polarized cell division (Abdelrahman et al., 2016; as reviewed by Ouellette et al., 2020) using host metabolites and energy to synthetize macromolecules. After multiple consecutive cycles of replication, RBs differentiate into EBs and are released from the host cell, *via* lysis or extrusion, to infect new host cells (AbdelRahman and Belland, 2005; Elwell et al., 2016). In women, cervical Ct infections can ascend to the upper genital tract, leading to serious sequelae, including pelvic inflammatory disease (PID), infertility, and ectopic pregnancy (EP) (Department of Health and Human Services, 2020). Mother-to-child transmission at birth can also cause conjunctivitis and/or pneumonia (Medline et al., 2017). In the United States (US), *Chlamydia* infection is the most common STI, with higher cases among adolescents. Cases of *Chlamydia* are higher in women ages 18 to 20 years and men ages 20 to 24 years. Women are infected 2.5 times more than men, possibly because of higher screening rates in women than men (Markle et al., 2013).

Ng, a Gram-negative diplococcus, initially establishes infection by adhering to host epithelial cells *via* bacterial surface structures – such as type IV pili, opacity (Opa) proteins, lipooligosaccharide (LOS), and the major outer membrane protein porin (PorB) (Quillin and Seifert, 2018). Upon adherence, Ng replicates and forms microcolonies and is also capable of host cellular invasion and transcytosis (Quillin and Seifert, 2018). Similar to Ct infection, untreated cervical Ng infection may progress to the upper reproductive tract, contributing to PID, infertility, and EP. Maternal transmission to children during birth can lead to neonatal blindness (Medline et al., 2017; Department of Health and Human Services, 2020). In the US, from 2014-2018, the reported Ng rate increased more than the reported Ct rate (63% vs 16%, respectively). Particularly dramatic was the Ng rate increase (137%) which occurred among men who had sex with men (MSM) and 51% of the Ng isolates tested were resistant to at least one antibiotic (Department of Health and Human Services, 2020).

Co-infections with Ct and Ng are common, with approximately 10%-40% of those infected with Ng also positive for Ct infection (Creighton et al., 2003). According to a study in 1998, women partners of Ct/Ng co-infected men showed a somewhat higher Ct infection rate (76%) than women partners of men singly infected with Ct (65%), while Ng infection rates were similar in women partners of Ct/Ng co-infected men and women partners of Ng singly infected men (73% vs 71%, respectively) (Lin et al., 1998). It has been suggested that gonorrhea may activate latent chlamydial infections (Richmond et al., 1972; Batteiger et al., 1989). This hypothesis was further supported by Lin et al. (1998), who noted that co-infection with Ng may have caused re-activation of latent or persistent Ct infection in women partners of Ng-only infected men. Additionally, recent evidence suggests that Ct/Ng co-infection may increase Ng transmission (Stupiansky et al., 2011). Furthermore, mathematical modelling indicates Ct/Ng co-infection occurs more often than expected due to chance, suggesting biological interaction between the two bacteria (Althaus et al., 2014). Despite the high rates of Ct/Ng co-infections detected clinically, *in vitro* and experimental animal model studies have focused on each pathogen individually. Thus, novel models are needed to explore co-infection (Leonard et al., 2019).

Recently, a *C. muridarum* (Cm)/Ng co-infection mouse model was established (Vonck et al., 2011). This model, in which BALB/c mice are infected first with Cm to model human Ct infection and then with Ng, showed increased vaginal shedding of Ng compared to the Ng-only-infected mice. In contrast, *in vitro* Cm pre-infection did not increase Ng adherence to, or invasion of, immortalized murine epithelial cells (Vonck et al., 2011). In the second and, to our knowledge, the only other *in vitro* study to address Ct/Ng co-infection, Ct-infected neutrophils were less able to kill Ng *via* neutrophil extracellular traps (NETs), increasing survival of Ng in neutrophils (Rajeeve et al., 2018). These two studies provide experimental evidence that biological interactions between the two pathogens may be present, with chlamydial infection potentially enhancing subsequent Ng infection *in vivo*. However, the reversed inoculation sequence (1^st^ Ng, 2^nd^ Cm or Ct) has not yet been investigated, either *in vitro* or in a mouse model.

Our aim was to determine if Ng co-culture influences chlamydial infection and development *in vitro*. We hypothesized that Ng-infected cells are more susceptible to chlamydial infection than uninfected cells. To facilitate this evaluation, we established an *in vitro* model of Ct/Ng co-infection in cultured cervical epithelial cells. We found that Ng co-culture negatively impacts chlamydial development—reducing inclusion number, inclusion size, and infectious EB production. Though this finding is not consistent with our hypothesis, it provides evidence that interaction of these bacteria *in vitro* influences the course of chlamydial development and infectivity. How such interactions may influence chlamydial development and infectivity *in vivo*, to date, remains largely unexplored.

To our knowledge, this is the first Ct/Ng co-infection model established in epithelial cells *in vitro*. This model has potential to contribute to understanding Ct/Ng pathogenesis and serve as a useful research tool to aid further *in vitro* and *in vivo* co-infection studies, which is a key step for informing ongoing treatment/prevention strategies for these two important STI agents.

## Materials and Methods

### Host Cells and Media

HeLa cells (ATCC^®^ CCL2™, human cervical adenocarcinoma epithelial cell line, American Type Culture Collection, Manassas, VA, USA; provided by Christian Blenn, University of Zurich), and LLC-MK2 (LLC) cells (Rhesus monkey kidney cell line; provided by IZSLER, Brescia, Italy) were grown at 37°C, 5% CO_2_ in culture medium. Culture medium consisted of: Dulbecco’s MEM (DMEM) Ham’s F-12 (dry), without Niacinamide, Tryptophan (US Biological, Salem, MA, USA) prepared per manufacturer’s recommendation and supplemented to final concentrations of 10% heat-inactivated fetal calf serum (FCS; BioConcept, Allschwil, Switzerland), 1.2 mg/ml Sodium Bicarbonate (7.5% sterile solution, Sigma-Aldrich/MilliporeSigma, St. Louis, MO, USA), 10-25 mM N-2-hydroxyethylpiperazine-N-2-ethane sulfonic acid (HEPES; 1 molar sterile solution, Sigma-Aldrich), 2 mM GlutaMAX-I (GlutaMAX Supplement, 100X; Gibco, Thermo Fisher Scientific, Waltham, MA, USA), 1% MEM non-essential amino acids (MEM NEAA; 100X, Gibco), 10 μg/ml L-Tryptophan (Alfa Aesar, Thermo Fisher Scientific), and 2.02 μg/ml Nicotinamide (Sigma-Aldrich/MilliporeSigma). See Supplementary file (Supplementary Text 2 - Media Composition) for detailed media components. Unless otherwise stated, cells were seeded in 24-well plates with sterile glass coverslips for microscopic analyses or without coverslips for sample collection for *Chlamydia trachomatis* serovar E (CtE) titration by sub-passage. Unless noted otherwise, HeLa were seeded at 3x10^5^ and LLC at 1.5x10^5^ cells/well in culture medium and after overnight culture, culture medium was supplemented with cycloheximide at 1 μg/ml and 1.5 μg/ml final concentrations for Hela and LLC, respectively.

### CtE Propagation

CtE (E/UW-5/CX) was originally obtained from S. P. Wang and C. C. Kuo (University of Washington) and is a genital tract isolate (Holt et al., 1967). CtE was propagated as previously reported in LLC cells (Marangoni et al., 2014), and crude stocks were suspended in a sucrose phosphate glutamate buffer (SPG; 218 mM sucrose (Sigma-Aldrich/MilliporeSigma); 3.76 mM KH_2_PO_4_ (Sigma-Aldrich/MilliporeSigma), 7.1 mM K_2_HPO_4_ (Sigma-Aldrich/MilliporeSigma), 5 mM GlutaMAX (Gibco, Thermo Fisher Scientific)) and stored at -80°C until use. Inclusion forming unit (IFU)/well determination was carried out in LLC and HeLa cells as previously described (Leonard et al., 2015; Onorini et al., 2019). Equivalent infection of HeLa, as compared to LLC, resulted in similar stock IFU/mL determination.

### Ng Propagation and CFU/ml Determination

Three laboratory strains of Ng were selected for model development: Ng strains NG (control type strain, ATCC 19424) and PPNG (penicillinase-producing strain, ATCC 31426) were purchased in Kwik-Stik format (Microbiologics, St. Cloud, MN, USA). Ng strain FA1090 (FA) (endocervical isolate, originally isolated from a probable disseminated infection; Cohen et al., 1994) was provided by Magnus Unemo, School of Medical Sciences, Orebro University. Ng strains were cultivated on commercially available chocolate agar (Thermo Scientific Chocolate agar with Vitox; Thermo Fisher Scientific). Ng stocks were frozen after 2 passages in our laboratory. Before each use, stock aliquots were thawed, cultured, and sub-passaged once on commercial chocolate agar prior to suspension and use (i.e., at passage 4). Note that, because penicillinase production/penicillin resistance of PPNG was not relevant to our study, PPNG was cultured on penicillin-free commercial chocolate agar (to improve PPNG growth, which is somewhat less robust on penicillin-containing agar).

For colony forming unit (CFU)/ml determination of Ng suspensions used in experiments, Ng colonies (18-24 h) were collected with a sterile nylon swab and suspended in sterile phosphate buffered saline (PBS, pH 7.2, 1 liter tablets; Canvax Biotech, Córdoba, Spain) to a McFarland density (McF) of 1.0 (DEN-1B densitometer, Grant Instruments, Cambridgeshire, UK; 0.5-4.0 McFarland Standard Set, Pro Lab Diagnostics, Richmond Hill, ON, Canada). Dilutions of these suspensions, made in sterile 0.05% saponin (Sigma-Aldrich/MilliporeSigma)/PBS, were cultured on Chocolate agar for 18-24 h. Bacterial colonies were counted on a stereo microscope (M3; Wild Heerbrug AG, Heerbrug, Switzerland) and CFU/ml of PBS suspension was calculated. McF of 1.0 consistently corresponded to 10^8^ CFU/ml for all three Ng strains used in the study. In each experiment, dilution and agar culture from PBS suspension was similarly performed to confirm the experiment-specific CFU/ml was as expected.

### Infections

#### Ng Infection

Immediately upon PBS suspension, further Ng dilutions were made in room temperature cycloheximide-supplemented culture medium to achieve the minimum CFU inocula required for Ng growth (PPNG 10^5^ CFU/ml, FA 10^4^ CFU/ml and NG 10^1^ CFU/ml; see Supplementary Text 1 - Supplementary Materials and Methods) in experiments without gentamycin or 10^6^ CFU in experiments with gentamycin, unless otherwise stated in the Figure legends. Immediately upon Ng media dilution, HeLa were infected with 1 ml/well of the prepared Ng media dilutions and 1 ml/well additional cycloheximide-supplemented culture medium was added.

#### CtE Infection

After 24 h of cycloheximide exposure (with or without Ng pre-infection, depending on experiment), cells (not DEAE-Dextran treated) were infected with 1 ml/well culture medium-diluted CtE stock; plates were centrifuged at 1000g, at 33°C for 1 hour. All CtE infections were carried out at MOI 0.225 and resulted in 15%-25% of infected cells. Immediately after CtE, inocula were replaced with cycloheximide-supplemented culture medium (with or without Ng post-infection, depending on experiment) and plates were incubated at 37°C in 5% CO_2_ for 40 h. Where noted, the cycloheximide-supplemented culture medium added immediately after CtE infection was further supplemented with 200 µg/ml gentamycin.

### Immunofluorescence (IF) Microscopy

At experimental endpoints, culture medium was removed from infected cells, cells were fixed with ice cold absolute methanol for 10 minutes and methanol was replaced with sterile PBS. *Chlamydiae* were immunostained as previously reported (Onorini et al., 2019) with some modification of antibody concentrations: 1:400-diluted *Chlamydiaceae* family-specific mouse monoclonal chlamydial anti-lipopolysaccharide (LPS) antibody (Clone ACI-P, Progen, Heidelberg, Germany) and 1:750-diluted Alexa Fluor 488-conjugated secondary goat anti-mouse antibody (Alexa 488; Life Technologies, Thermo Fisher Scientific). Host and chlamydial DNA were stained with 1 μg/ml 4’, 6-diamidino-2’-phenylindole dihydrochloride (DAPI); Molecular Probes, Thermo Fisher Scientific). Coverslips mounted on glass slides using FluoreGuard mounting medium (Hard Set; ScyTek Laboratories Inc., Logan, UT, USA) were evaluated using a Leica DMLB fluorescence microscope (Leica Microsystems, Wetzlar, Germany). Representative micrographs were generated using BonTec software (BonTec, Bonn, Germany) and a UI-2250SE-C-HQ camera (uEye, IDS Imaging Development Systems GmbH, Obersulm, Germany).

HeLa nuclei and chlamydial inclusions were counted in randomly selected, reticle-delimited, microscopic fields at 1000X magnification (100X oil immersion objective, 10X ocular, and corresponding imaging adjustment). To determine mean inclusion size (expressed as inclusion area, μm^2^), randomly selected inclusions were examined at 400X (40X objective, 10X ocular, and corresponding imaging adjustment) magnification and area (μm^2^) were calculated for each inclusion, using BonTec measuring and archiving software (BonTec, Bonn, Germany) as previously described (Onorini et al., 2019).

### CtE Titration by Sub-Passage

For titration by sub-passage, infected monolayers were scraped into the 2 ml of culture medium in the sample well and stored at -80°C until titrated in LLC cells. Samples were thawed, vortexed briefly, serially diluted in culture medium, and used to infect duplicate wells of LLC cells seeded on glass coverslips. Gentamycin (200 µg/ml) was included during incubation to prevent Ng growth. Fixation, immunostaining and microscopic analysis was performed as described for IF microscopy. The number of inclusions per field was determined and IFU/well was calculated as previously described (Onorini et al., 2019).

### Study Design and Statistical Analysis

Biological and technical replicates are defined here as per Pollard et al. (Pollard et al., 2019). Namely, biological replicates represent different samples, while technical replicates represent multiple measurements made from a single sample. Per an independent experiment, we prepared two biological replicates for each experimental group (i.e., two different samples prepared as two different wells); 30-50 technical replicate measurements per biological replicate were made for microscopy-measured assays (30 measurements for nuclei/well, inclusions/well, and IFU/well; 50 measurements for inclusion size), and 3 technical replicate measurements were made for agar plate-based assays (determination of Ng CFU/ml in culture). Sample coding was routinely used to blind sample identification prior to evaluation.

Unless otherwise stated in the Figure legends, n=6 (6 biological replicates from 3 independent experiments) and data represent mean values ± standard deviation of biological replicates, calculated with Microsoft Excel (Microsoft, Redmond, WA, USA). Statistical analyses were performed using R software (R Core Team). Significance of the difference of means was determined by ANOVA and means of each Ng group were further compared to the Ct control mean by Dunnett’s *post hoc* test. P values of <0.05 were considered significant (*p<0.05; **p<0.01;***p<0.001).

## Results

### Establishing a Co-Infection Model: Conditions for Ng and CtE Co-Infection

To investigate the potential impact of Ng co-infection on CtE development and infectivity *in vitro*, we sought to develop an STI co-infection model system to allow evaluation of chlamydial inclusion size (expressed as inclusion area, μm^2^) and inclusion number as measures of chlamydial development and evaluation of infectious EB production as a measure of chlamydial infectivity. To achieve this, experimental conditions were required to support the growth of both bacterial species. Additionally, because *Chlamydia* is an obligate intracellular bacterium, conditions were also required to control and support host cell viability for the approximate duration of the chlamydial developmental cycle to allow observation of potential Ng-specific impact on chlamydial development, as opposed to non-specific limitation of chlamydial growth caused by destruction of the host cell monolayer. Supplemented DMEM/F12 cell culture medium (see Supplementary Text 1 - Supplementary Materials and Methods and Supplementary Text 2 - Media Composition) was selected because it is appropriate for genital and intestinal epithelial cells, such as the HeLa cervical epithelial cell line, typically used for the study of various chlamydiae (Moore et al., 2008; Pettengill et al., 2012). DMEM has also been previously reported to support Ng culture and Ng/*Lactobacillus* co-culture (Jones et al., 2007; Spurbeck and Arvidson, 2008).

To determine equivalent and sufficient inocula of the Ng strains evaluated, we determined colony forming units (CFU)/ml for phosphate buffered saline (PBS) suspensions from 18-22 h in chocolate agar cultures. In preliminary experiments, all Ng strains grew in the culture medium but the minimum colony forming units (CFU) of inoculum required to consistently result in Ng survival and growth in the medium varied considerably across the strains, which we considered to be characteristic of each strain. To ensure Ng survival/growth in culture and to standardize the Ng inocula based on culture medium viability, we used 10^1^ higher CFU than the minimum Ng inocula required for consistent growth in culture medium: 10^5^ CFU for PPNG, 10^4^ CFU for FA, and 10^1^ CFU for NG (see Supplementary Text 1 - Supplementary Materials and Methods). These Ng inocula are referred to here as the “minimum” Ng inocula required for growth in culture medium.

Initially, HeLa cells were seeded at low density to reach approximate confluence at the experimental endpoint and were CtE-infected in the absence of cycloheximide exposure and using crude stocks, rather than purified EB, to represent the natural infection process. Inoculation with any of the Ng strains, 24 h before CtE or mock infection, caused substantial host cell death and monolayer loss compared to uninfected and CtE-infected controls (**Supplementary Figure 1**). This cell death was observed regardless of whether gentamycin was added at the time of CtE infection to limit continued Ng growth. Chlamydial inclusion size was dramatically reduced by Ng co-infection in the absence of gentamycin exposure (**Supplementary Figure 1**). Gentamycin exposure abolished Ng-dependent reduction in inclusion size and inclusions were instead, larger than corresponding CtE-infected controls (**Supplementary Figure 1**). However, heavy cell loss (ranging from 20%-70% of host cells lost) occurred with Ng single infection or CtE co-infection and thus, increased inclusion size might have resulted from decreased host cells numbers and corresponding increased culture area available per cell and thus, cannot be clearly attributed to Ng co-infection. Additionally, though Ng-infection generally reduced inclusions per well and infectivity (IFU/well), Ng-dependent cell loss confounded potential analysis of Ng-dependent effects on inclusion numbers and infectivity **(Supplementary Figure 1**). Finally, excessive host cell loss grossly disturbed potential for the model system to support similar/equivalent chlamydial development in control versus Ng-infected groups over the course of the typical complete chlamydial developmental cycle (40 h for CtE).

**Figure 1:**
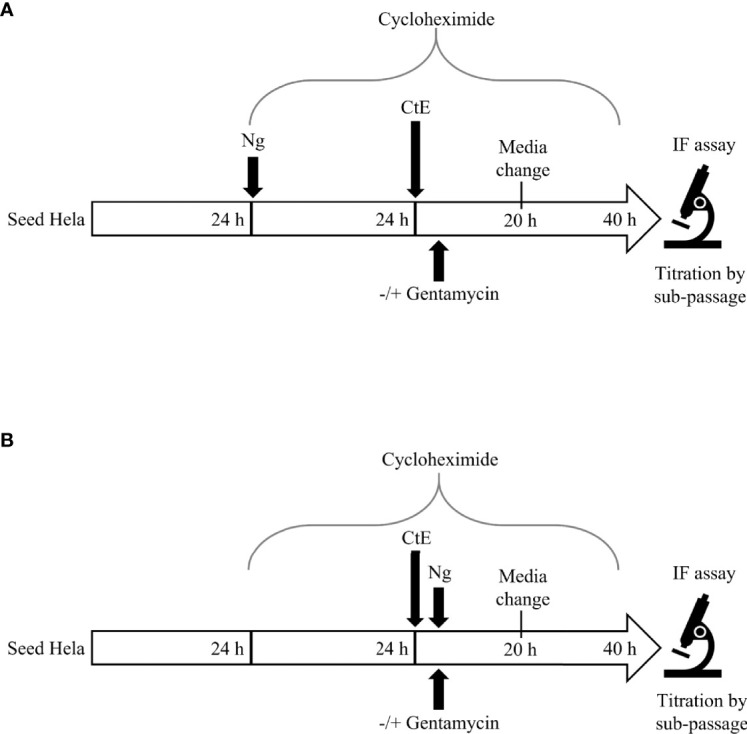
Experimental Design. For Ng pre-infection **(A)**, 24 h after seeding in 24 well plates, HeLa cells were infected with Ng in cycloheximide-supplemented culture medium for 24 h prior to subsequent CtE infection. Immediately after CtE infection, inocula were replaced with fresh cycloheximide-supplemented culture medium without gentamycin, to allow continued Ng growth (**Figure 2**), or with gentamycin, to restrict further Ng growth (**Figure 4**). For Ng infection after CtE infection **(B)**, 24 h after seeding in 24 well plates, HeLa cells were exposed to cycloheximide-supplemented culture medium (no Ng) for 24 h prior to subsequent CtE infection. Immediately after CtE infection, inocula were replaced with Ng in fresh cycloheximide-supplemented culture medium without gentamycin, to allow Ng growth (**Figure 3**), or with gentamycin, to restrict Ng growth (**Figure 5**). In both experimental settings **(A, B)**, cycloheximide-supplemented medium was refreshed at 20 h post CtE infection, with gentamycin concentrations maintained, and samples were processed for IF analysis of inclusion development/formation or determination of infectivity (IFU/well) at 40 h post-CtE infection.

Our initial studies (**Supplementary Figure 1**) clearly indicated that the co-infection culture conditions needed to be altered to support host cell viability and eliminate host cell loss, so that observed effects of Ng on Ct development could be attributed to direct Ng effects rather than indirect effects of host cell loss. Thus, host cells were seeded and cycloheximide was exposed the following day to limit ongoing cell division during the course of the experiment. This modification allowed continuous co-culture of HeLa cells and all tested Ng strains for 24 h prior to CtE infection, and/or for the subsequent duration of the complete CtE developmental cycle (**Figure 1**), without host cell death/loss (**Supplementary Figure 2**). Additionally, *in situ* trypan blue staining of replicate monolayers (see Supplementary Text 1 - Supplementary Materials and Methods) demonstrated that Ng pre-infection did not compromise host cell plasma membrane integrity, as compared to CtE singly-infected cells in any subsequent experiment (**Supplementary Figure 3**). As expected (Brodeur et al., 1977), cycloheximide exposure, even at up to 10-15 times the concentration used in these experiments, did not limit Ng growth in DMEM/F12 medium culture (not shown) and centrifugation-assisted chlamydial infection was adopted to improve chlamydial infection rate in all subsequent experiments.

### Pre-Infection of Host Cells With Ng Limits CtE Inclusion Development and Infectivity

To determine if Ng pre-infection alters subsequent CtE infection (**Figure 1A**), HeLa cells were infected with the minimum inoculum of each Ng strain (PPNG, FA, NG) in cycloheximide-supplemented medium or with cycloheximide-supplemented medium alone as a control. After 24 h, turbid growth of all Ng strains in inoculated wells were clearly visible by eye and by light microscopy. The Ng-infected or uninfected cells were then infected with CtE and the cultures were incubated for 40 h (**Figure 1A**). Turbid growth of Ng was again visible at the 20 h medium change and at the end of the experiment.

**Figure 2:**
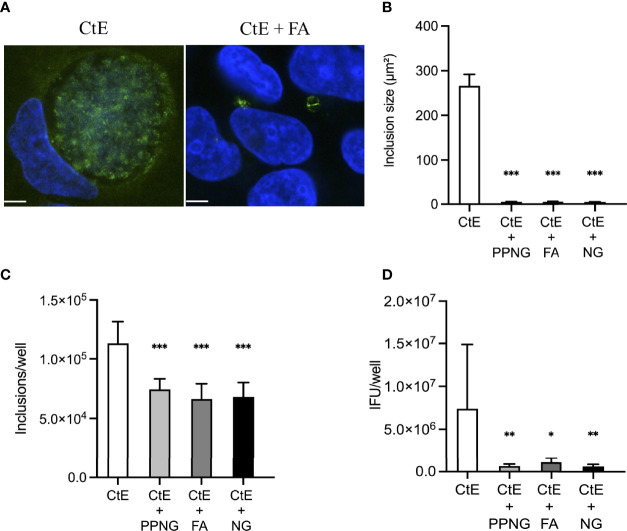
Pre-infection of host cells with Ng limits CtE inclusion development and infectivity. HeLa cells were infected with Ng (minimum CFU inocula required for growth in culture medium) in cycloheximide-supplemented culture medium for 24 h prior to subsequent CtE infection (**Figure 1A**). Immediately after CtE infection, inocula were replaced with fresh cycloheximide-supplemented culture medium. Cycloheximide-supplemented culture medium was refreshed at 20 h post-CtE infection and samples were processed for IF analysis of inclusion formation/development or determination of infectivity (IFU/well) at 40 h post-CtE infection. Representative micrographs **(A)** of CtE (left) and CtE + FA (right) experimental groups, CtE inclusion size **(B)** (expressed as inclusion area, μm^2^), CtE inclusions/well **(C)**, and CtE IFU/well **(D)** are shown. Scale bars = 5 µm; DAPI staining of DNA is indicated in blue and LPS immunolabeling of CtE is indicated in green. Significant differences from the control are indicated with asterisks: *p < 0.05, **p < 0.01, ***p < 0.001.

Pre-infection for 24 h with PPNG, FA, or NG reduced CtE inclusion size (**Figure 2A**), with average sizes of 4.83 ± 0.47 μm², 5.22 ± 0.80 μm² and 4.52 ± 0.28 μm², respectively, compared to 266.27 ± 25.71 μm² for the control (CtE only), representing reduction to less than 2% of control inclusion size (**Figure 2B**). Moreover, Ng pre-infection reduced the number of chlamydial inclusions (**Figure 2C**) by 34%-40% (7.44x10^4^ ± 8.92x10^3^ inclusions/well for PPNG, 6.63x10^4^ ± 1.29x10^4^ inclusions/well for FA, 6.81x10^4^ ± 1.21x10^4^ inclusions/well for NG) compared to control CtE singly-infected cells (1.13x10^5^ ± 1.83x10^4^ inclusions/well). A corresponding reduction in the production of infectious chlamydiae was observed: 91% reduction in titer by PPNG (6.59x10^5^ ± 2.40x10^5^ inclusions forming unit (IFU)/well), 85% reduction by FA (1.11x10^6^ ± 5.02x10^5^ IFU/well), and 92% reduction by NG (6.23x10^5^ ± 2.46x10^5^ IFU/well), as compared to the control (7.40x10^6^ ± 7.48x10^6^ IFU/well) (**Figure 2D**). Taken together, these data demonstrate that a pre-infection with Ng neither kills nor prevents the host cells from supporting subsequent CtE infection, nor does it strictly abolish the ability of CtE to infect host cells. However, Ng-pre-infection limited inclusion formation and development and production of infectious progeny, suggesting that, in this experimental setting, Ng exerts an anti-chlamydial effect.

### Reduced Medium pH Alone Is Not Sufficient to Cause Ng-Pre-Infection-Dependent Anti-Chlamydial Effects on CtE

Decreased culture medium pH, as estimated by medium phenol red coloration, was noted at the times of CtE infection, medium change, and experimental endpoint for Ng pre-infected wells compared to the CtE singly infected wells (not shown). A possible explanation for the observed effects of Ng-pre-infection is this acidification of the medium. To address this, we evaluated the effect of reduced culture medium pH on CtE inclusion development and infectivity in the absence of Ng infection using media adjusted to various pH (pH 7.4, 7.0, 6.5 or 6.0; see Supplemental Text 1 - Supplemental Materials and Methods). These media were maintained on cells after CtE infection and refreshed at 20 h post CtE-infection, to simulate the pH effects of Ng infection. Medium of pH 7.0 or 6.5 did not affect host cell numbers or measures of CtE development (**Supplementary Figures 4A–D**). In contrast, exposure to culture medium of pH to 6.0 reduced CtE inclusion size and infectivity, as well as host cell numbers (**Supplementary Figures 4A–D**), illustrating that reduced pH can have a negative effect on CtE inclusion development and infectivity in this experimental setting. However, reduction of culture medium below pH 6.5 was not observed in the course of our experiments, suggesting that reduction of the extracellular culture medium pH is not responsible for the observed Ng-dependent anti-chlamydial effects.

### Media Nutrient Supplementation Does Not Alleviate Ng-Dependent Anti-Chlamydial Effects on CtE

Because Ng grew to high turbidity in our model, we considered that the Ng-specific anti-chlamydial effects might be due, in part, to extracellular media nutrient depletion. To evaluate this possibility, we performed a 24 h pre-infection of HeLa with FA (selected as more relevant as it is widely used in *in vitro* and mouse studies) in supplemented media with increased concentrations of glucose, essential (other than glutamine) and non-essential amino acids, glutamine, or combined glucose/amino acid/glutamine (see Supplemental Text 1 - Supplemental Materials and Methods). These supplemented media (pH 7.4), were included throughout the course of the experiment with supplementation maintained after CtE infection and refreshed 20 h after CtE infection. In the absence of FA, individual supplementation increased CtE infectivity (IFU/well) by 60%-90% compared to un-supplemented medium, while combined supplementation reduced CtE infectivity by 45% (**Supplementary Figure 5**). However, individual or combined media supplementation did not alleviate FA-dependent anti-chlamydial effects (**Supplementary Figure 5**), suggesting that the observed effects of Ng do not depend on depletion of these nutrients from the extracellular cell culture medium.

### Ng Infection Immediately After CtE Infection Limits Chlamydial Inclusion Development and Infectivity

In the course of the above-described Ng 24 h pre-infection of HeLa cells prior to subsequent CtE infection, total duration of Ng infection was 64 h, and robust Ng growth in the culture medium, observed as visible turbidity, was present both before and throughout CtE infection and development. Media change/replenishment at CtE infection and the subsequent media change 20 h after infection thus resulted in variable Ng load in the media over time but did not eliminate Ng infection of the cells/media, such that live Ng were present at the time of CtE infection. To determine if Ng infection must occur *prior* to CtE infection to exert the observed anti-chlamydial effects, we inoculated CtE-infected cells with Ng (minimum inocula for liquid culture) in cycloheximide-supplemented media added immediately after CtE infection (**Figure 1B**). Cells were exposed to cycloheximide for 24 h prior to CtE infection to maintain the same duration of cell culture and cycloheximide exposure as the 24 h Ng pre-infection experiments (**Figure 1A**).

**Figure 3:**
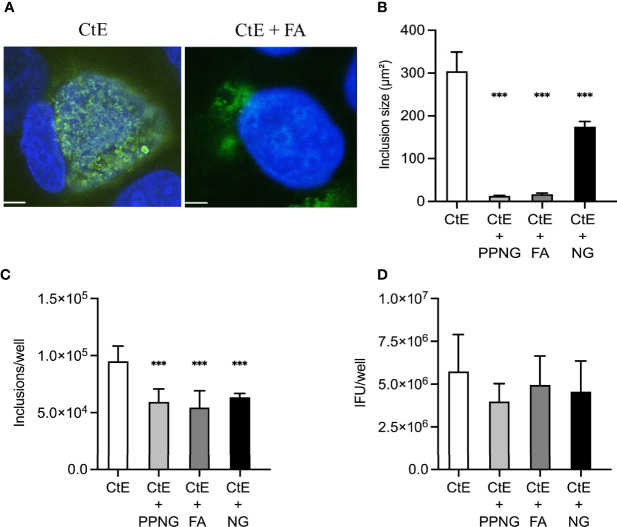
Ng infection immediately after CtE infection limits chlamydial inclusion development. HeLa cells were exposed to cycloheximide-supplemented culture medium (no Ng) for 24 h prior to subsequent CtE infection. Immediately after CtE infection, cells were infected with Ng (minimum CFU inocula required for growth in culture medium) in fresh cycloheximide-supplemented culture medium (**Figure 1B**). Cycloheximide-supplemented culture medium was refreshed at 20 h post-CtE infection and samples were processed for IF analysis of inclusion formation/development or determination of infectivity (IFU/well) at 40 h post-CtE infection. Representative micrographs **(A)** of CtE (left) and CtE + FA (right) experimental groups, CtE inclusion size **(B)** (expressed as inclusion area, μm^2^), CtE inclusions/well **(C)**, and CtE IFU/well **(D)** are shown. Scale bars = 5 µm; DAPI staining of DNA is indicated in blue and LPS immunolabeling of CtE is indicated in green. Significant differences from the control are indicated with asterisks: *p < 0.05, **p < 0.01, ***p < 0.001.

In this experimental setting, PPNG and FA infection reduced CtE inclusion size (**Figure 3A**) to less than 6% of the control CtE inclusion size (12.70 ± 1.56 μm² and 16.80 ± 2.72 μm² respectively; CtE, 304.01 ± 45.21 μm²). The NG strain reduced inclusion size (174.56 ± 12.23 μm²) to 57% of the control value, an effect notably less pronounced and observed for the other two Ng strains (**Figure 3B**). Moreover, subsequent infection of CtE-infected cells with each of the three Ng strains reduced inclusion number to 57%-67% of that in the CtE-only control (CtE + PPNG, 5.94x10^4^ ± 1.14x10^4^ inclusions/well; CtE + FA, 5.44x10^4^ ± 1.48x10^4^ inclusions/well; CtE + NG, 6.36x10^4^ ± 3.28x10^3^ inclusions/well; CtE-only, 9.51x10^4^ ± 1.33x10^4^ inclusions/well) (**Figure 3C**).

The Ng inocula, referred to here as “minimum” Ng inocula, required for liquid culture, resulted in growth to 10^8^ CFU/ml (range = 9.8x10^7^ to 1.8x10^8^ CFU/ml) after 24 h of culture with HeLa cells in culture medium, as did a single higher inoculum of 10^6^ CFU for each Ng strain (range = 8.4x10^7^ to 2.9x10^8^ CFU/ml) (**Supplementary Figure 6**). However, the NG strain, specifically, with the lowest minimum inoculum of 10^1^CFU, showed the most robust increase in CFU/ml with a higher versus lower CFU inoculum (increase of 50% from 1.8x10^8^ to 2.9x10^8^ CFU/ml, resulting from inocula of 10^1^ and 10^6^, respectively; **Supplementary Figure 6**). This suggested that increasing NG strain inoculum might increase the experimental CFU/ml reached, and potentially cause a more robust reduction of CtE inclusion size, similar to that observed for PPNG and FA. Therefore, we repeated CtE-alone versus CtE + NG co-infection using a higher 10^6^ CFU NG inoculum. A higher CFU NG strain inoculum had a more pronounced effect on CtE inclusion size than on CtE inclusion number (**Supplementary Figure 7**). These results taken together, indicate that all three Ng strains have the capacity to markedly reduce CtE inclusion size and inclusion number when co-infecting HeLa/CtE, whether Ng infection occurs prior to, or immediately after, CtE infection.

Ng infection, either with minimum inocula required for liquid culture or with 10^6^ CFU, prior to or immediately after CtE infection, coincided with both culture medium turbidity and pronounced association of Ng with the entire host cell monolayer. This could be observed by light microscopy (not shown) throughout the experiments, even after inoculation with CtE and replacement of CtE inoculum with culture medium and at 20 h and 40 h post-CtE infection. This indicated that robust Ng growth before and throughout CtE infection/development coincides with intimate association of Ng to HeLa cells. Sequential immunostaining, for CtE then Ng, confirmed that FA was associated with all cells in the host monolayer in both Ng single-infected and Ct/Ng co-infected cells (**Supplementary Figure 8**). This result thus also indicates that both CtE-infected as well as uninfected HeLa cells can serve as targets for Ng adherence/colonization.

### Reduced Media pH *Immediately After* CtE Infection Increases CtE Infectivity, Potentially Masking Ng-Dependent Anti-Chlamydial Effect on Infectivity

Unexpectedly, given the observed marked reductions in inclusion size/number, corresponding reduction in infectivity was not observed for any of the three Ng strains when they were inoculated immediately after CtE infection (**Figure 3D**). This was a surprising finding given the observed reduction in inclusion size and inclusion numbers across the three Ng strains, compared to the CtE-only control (**Figures 3A–C**). This was also particularly unexpected considering that, in the setting of 24 h pre-infection with Ng, similar reductions in inclusion size and number were associated with reduction to 8%-15% of control infectivity (**Figure 2**).

The impact of culture medium pH was again evaluated (see **Supplemental Text 1** - Supplemental Materials and Methods). To simulate pH changes associated with Ng infection after CtE infection, pH adjusted medium was applied to CtE-infected HeLa cells, minus Ng infection, exactly as described in section 3.3, except pH modulation was not initiated until after CtE infection. In contrast to exposure to lower pH culture media initiated 24 h *prior* to CtE infection (**Supplementary Figures 4A–D**), which did not influence CtE infectivity, exposure to media with pH 7.0 or 6.5 initiated immediately *after* CtE infection was associated with a marked 100% *increase* in CtE infectivity compared to pH 7.4 media. Notably, this increase in infectivity was not accompanied by a concomitant increase in inclusion size or number (**Supplementary Figures 4E–H**). Similar to 24 h Ng pre-infection of HeLa/CtE, inoculation with Ng after CtE infection resulted in a decrease of culture medium pH to 6.5-7.0 at the time of the media change 20 h later (not shown). Because Ng-infected groups, but not the CtE-only control, have reduced medium pH, potential Ng-mediated reduction in CtE infectivity may be blunted or masked by pH-dependent increased infectivity in the Ng-co-infected groups *specifically when Ng is inoculated after the CtE infection*.

### Infection of HeLa/CtE With Increased Ng Inoculum, Immediately After CtE Infection, Overcomes the Putative General Lower-pH-Specific Increase in Chlamydial Infectivity

This raised the question of whether increasing the Ng inocula might also increase the anti-chlamydial effect on chlamydial *infectivity* when inoculated immediately after chlamydial infection. We repeated the experiment, replacing the minimum required FA inoculum for growth in culture medium (10^4^ CFU) with a higher dose of 10^6^ CFU of FA. The pH of the culture media in the CtE + FA group was again reduced by FA growth to only pH 6.5-7.0, as previously seen with 10^4^ FA inoculum (**Figures 3B, C**; **Supplementary Figure 2**). CtE inclusion size and number were reduced in the CtE + FA group, as compared to the CtE-only control (**Supplementary Figures 9A–C**). However, 10^6^ FA inoculum reduced CtE infectivity 82% compared to the control value (**Supplementary Figure 9D**). In contrast, the 10^4^ FA inoculum decreased CtE infectivity only 14% in the same experimental setting (**Figure 3D**). This indicates that FA infection can reduce infectivity, as well as inclusion size, when FA inoculum is increased, suggesting higher Ng inoculum can elicit an anti-chlamydial effect sufficient to overcome the opposing infectivity-enhancing effect of lowered media pH in this experimental setting.

### Gentamycin Exposure After Pre-Infection of Host Cells With Ng Reduces Ng-Dependent Anti-Chlamydial Effects on CtE

As previously noted, when HeLa cells were pre-infected with Ng 24 h prior to subsequent CtE infection, anti-chlamydial effects on inclusion formation/development and infectivity were observed (**Figure 2**). To determine if continued Ng viability after 24 h Ng pre-infection were needed for the obtained results, we exposed Ng pre-infected HeLa/CtE (10^6^ CFU inocula of all Ng strains) to gentamycin to restrict the duration of Ng growth to the 24 h prior to CtE infection (**Figure 1A**). Gentamycin was included in the medium after CtE infection and maintained for the duration of CtE culture, including at the media change at 20 h after CtE infection (**Figure 1A**), with the expectation that any intracellular live Ng exiting the host cells would be killed upon egress or shortly after (Lu et al., 2019).

**Figure 4:**
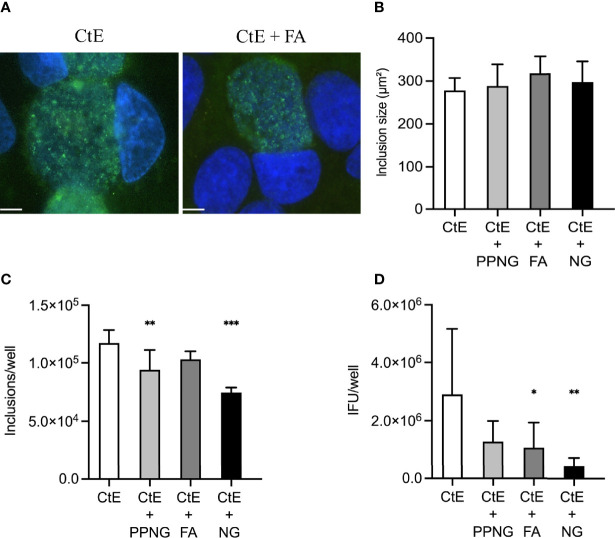
Gentamycin exposure after pre-infection of host cells with Ng limits Ng-dependent anti-chlamydial effects on CtE inclusion development and infectivity. HeLa cells were infected with Ng (10^6^ CFU/well) in cycloheximide-supplemented culture medium for 24 h prior to subsequent CtE infection. Immediately after CtE infection, inocula were replaced with fresh cycloheximide- and gentamycin-supplemented culture medium (**Figure 1A**). Cycloheximide- and gentamycin-supplemented culture medium was refreshed at 20 h post-CtE infection and samples were processed for IF analysis of inclusion formation/development or determination of infectivity (IFU/well) at 40 h post-CtE infection. Representative micrographs **(A)** of CtE (left) and CtE + FA (right) experimental groups, CtE inclusion size **(B)** (expressed as inclusion area, μm^2^), CtE inclusions/well **(C)**, and CtE IFU/well **(D)** are shown. Scale bars = 5 µm; DAPI staining of DNA is indicated in blue and LPS immunolabeling of CtE is indicated in green. Significant differences from the control are indicated with asterisks: *p < 0.05, **p < 0.01, ***p < 0.001.

Gentamycin exposure entirely abolished Ng-dependent reduction in CtE inclusion size (**Figures 4A, B**). In contrast, gentamycin exposure limited but did not entirely abolish Ng-dependent reduction in inclusion number to 64%-88% of control values (CtE + PPNG 9.41x10^4^ ± 1.69x10^4^ inclusions/well; CtE + FA 1.03x10^5^ ± 6.93x10^3^ inclusions/well; CtE + NG 7.46x10^4^ ± 4.33x10^3^ inclusions/well; CtE-only control value of 1.17x10^5^ ± 1.12x10^4^ inclusions/well) (**Figure 4C**). Additionally, gentamycin exposure attenuated, but did not abolish, Ng-dependent reduction of infectivity to 15%-44% of control values (CtE + PPNG 1.28x10^6^ ± 7.13x10^5^ IFU/well; CtE + FA 1.07x10^6^ ± 8.68x10^5^ IFU/well; CtE + NG 4.34x10^5^ ± 2.79x10^5^ IFU/well; CtE-only control value of 2.90x10^6^ ± 2.25x10^6^ IFU/well) (**Figure 4D**). As expected, when gentamycin was added, preventing subsequent Ng growth, the pH reduction previously observed was not present (not shown).

### Exposure to Gentamycin Concomitant With Ng Infection Immediately After CtE Infection, Limits Ng-Dependent Reduction of CtE Inclusion Size and Number and Abolishes Reduction of CtE Infectivity

**Figure 5:**
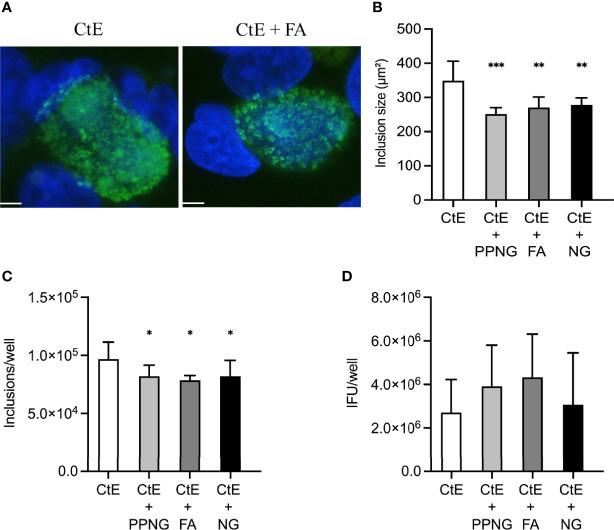
Exposure to gentamycin concomitant with Ng infection immediately after CtE infection limits Ng-dependent reduction of CtE inclusion size and inclusion number and abolishes Ng-dependent reduction of CtE infectivity. HeLa cells were exposed to cycloheximide-supplemented culture medium (no Ng) for 24 h prior to subsequent CtE infection. Immediately after CtE infection, cells were infected with Ng (10^6^ CFU/well) in fresh cycloheximide- and gentamycin-supplemented culture medium (**Figure 1B**). Cycloheximide- and gentamycin-supplemented culture medium was refreshed at 20 h post-CtE infection, and samples were processed for IF analysis of inclusion formation/development or determination of infectivity (IFU/well) at 40 h post-CtE infection. Representative micrographs **(A)** of CtE (left) and CtE + FA (right) experimental groups, CtE inclusion size **(B)** (expressed as inclusion area, μm^2^), CtE inclusions/well **(C)**, and CtE IFU/well **(D)** are shown. Scale bars = 5 µm; DAPI staining of DNA is indicated in blue and LPS immunolabeling of CtE is indicated in green. Significant differences from the control are indicated with asterisks: *p < 0.05, **p < 0.01, ***p < 0.001.

To evaluate if Ng inoculation immediately after CtE infection may still reduce measures of chlamydial development when active Ng growth is prevented, we initiated gentamycin exposure concomitantly with Ng inoculation immediately after CtE infection (**Figure 1B**). Previously, when Ng was added immediately after CtE, with no gentamycin, a strong decrease in inclusion size and inclusion number was observed, without a negative impact on chlamydial infectivity (**Figure 3**). When gentamycin was included in this setting, a decrease in inclusion size and inclusion number was still observed, even though it was not as robust as that without gentamycin (**Figures 5A–C**). However, infectivity was not significantly different from the control upon addition of gentamycin-exposed Ng (**Figure 5D**). These results indicate that Ng inoculated concomitantly with gentamycin immediately after CtE infection may still reduce chlamydial inclusion size and number but does not change infectivity.

This lack of decreased infectivity, given the slight but significant decrease in inclusion size and number observed in this setting, was unexpected. However, comparison of the infectivity of CtE-only controls specifically between gentamycin-excluding experiments and gentamycin-containing experiments revealed a gentamycin-dependent 50%-60% decrease in infectivity of the CtE-only controls (**Supplementary Figure 10D**). Notably, despite this marked gentamycin-dependent reduction of CtE infectivity, no similar effect was seen on CtE inclusion size or number (**Supplementary Figures 10B, C**) or host cell number (**Supplementary Figure 10A**). Thus, when Ngs are inoculated concomitantly with gentamycin, Ng-dependent amelioration of gentamycin-dependent reduction in chlamydial infectivity cannot be ruled out.

### Killed Ng or Ng Lysates Do Not Show Anti-Chlamydial Effect

To further evaluate if Ng viability is required for Ng anti-chlamydial effects, as suggested by abrogation of Ng anti-chlamydial effects by gentamycin exposure, we performed experiments with killed FA. FA was killed by exposure to either gentamycin or formalin, which preserves surface structures (see Supplemental Text 1 - Supplemental Materials and Methods; Patrone et al., 2006; Chao and Zhang, 2011). Washed and re-suspended killed FA, equivalent to 10^8^ CFU live FA per well, added either 24 h prior to CtE infection or immediately after CtE infection, and maintained throughout experiments, had no notable effect on CtE development/infectivity (not shown), in line with our previous gentamycin-exposure results, supporting that Ng viability is required for the observed Ng anti-chlamydial effects.

Additionally, similar exposure to FA lysate, equivalent to 10^6^ CFU live FA per well (see Supplemental Text 1 - Supplemental Materials and Methods), failed to reduce inclusion size/number and/or infectious EB production in either experimental setting (not shown), suggesting that FA cytosolic factors are not sufficient to cause the observed anti-chlamydial effects. Killed FA or FA lysate, as expected, had no effect on medium pH as estimated by medium phenol red coloration (not shown).

### Conditioned-Media may Contain Ng Factors With Anti-Chlamydial Effect(s) That Overcome pH-Dependent Increased CtE Infectivity

Finally, we evaluated the potential for host- or bacteria-secreted factors associated specifically with active Ng growth in the co-infection system to modulate chlamydial infection/infectivity. Filter-sterilized conditioned cycloheximide-containing media were generated/collected from 24 h cultures of: uninfected HeLa (no pH change), HeLa/CtE (no pH change), HeLa/FA and HeLa/CtE/FA (both = pH reduced to 6.5-7.0 by FA growth with HeLa, as previously mentioned), and compared to FA grown in medium alone or medium alone (controls, both no pH change, i.e., pH 7.4) (see Supplementary Text 1 - Supplementary Materials and Methods). No effect was observed on chlamydial inclusion size/number when any of the conditioned media were added 24h before CtE infection and maintained for the duration of the experiment (Supplementary **Figures 11A–C**). However, a 50% increase in infectivity was observed in the conditioned media made from HeLa cells only, suggesting that cycloheximide-exposed host cells may secrete some factor(s) which support CtE infectivity (**Supplementary Figure 11D**). As previously noted for pH-modified media applied to cells 24 h prior to CtE infection, media pH of 6.5-7.0 (as observed in the conditioned media of HeLa/FA and HeLa/CtE/FA) was not associated with modulation of CtE infectivity when cells were exposed *prior* to subsequent CtE infection, despite the renewal of media after CtE infection (**Supplementary Figure 4D**). Notably, in contrast to increased CtE infectivity caused by HeLa conditioned media, 24 h exposure to HeLa/FA conditioned media for 24 h prior to CtE infection, with subsequent maintenance of the conditioned media for the duration of the experiment, caused little to no change in CtE infectivity (**Supplementary Figure 11D**), suggesting that FA growth can potentially compete for/eliminate CtE-infectivity-enhancing secreted host cell factors or otherwise dampen the CtE-infectivity-enhancing effect of HeLa conditioned media in this experimental setting.

When conditioned media were added immediately *after* CtE infection and maintained for the duration of the CtE infection, there was, again, little/no effect on inclusion size/number when any of the conditioned media were added at the time of CtE infection (**Supplementary Figures 11E–G**). In contrast, when the HeLa/FA conditioned media, which has pH of 6.5-7.0, was added immediately after CtE infection, a marked increase in infectious EB production was again seen (**Supplementary Figure 11H**), producing similar results to those observed upon exposing CtE infected cells to media adjusted to pH 6.5-7.0 immediately after infection (**Supplementary Figure 4H**). However, HeLa/CtE/FA conditioned medium also had lowered pH of 6.5-7.0 but failed to increase CtE infectivity (**Supplementary Figure 11H**), indirectly suggesting that conditioned-media from CtE/FA co-infected cells may contain Ng factors with anti-chlamydial effect(s) that overcome pH-dependent increased CtE infectivity.

### Ng-Dependent Anti-Chlamydial Effect Is Not Restricted to Centrifugation-Assisted Chlamydial Infection, a Specific Host Cell Line, or Chlamydial Species

During model development, we evaluated the effects of Ng, in both experimental settings, in the context of cycloheximide-supplemented media with non-centrifugation assisted CtE infection, to better represent a natural infection. The observed Ng anti-chlamydial effect off all three Ng strains was centrifugation-independent (not shown). To determine whether the anti-chlamydial effects of Ng co-infection are host cell type specific, the experiments described in **Figure 1A** were repeated using the intestinal human epithelial cell line T84 and all three Ng strains and results were similar to those for HeLa cells (not shown). Finally, we evaluated the effect of FA on *C. muridarum* (Cm) development in HeLa cells, to consider potential impact of chlamydial species in both experimental settings (**Figures 1A, B**), with total chlamydial infection time reduced to 24 h (and medium change thus eliminated) due to the faster Cm developmental cycle. Cm results were similar to CtE results (not shown). Together these evaluations suggest Ng has a general anti-chlamydial effect.

## Discussion

Ct/Ng co-infections are common (Creighton et al., 2003), may be associated with increased Ng transmissibility (Stupiansky et al., 2011), could promote acquisition of Ng drug resistance (Unemo and Shafer, 2014), and interaction between the two organisms could cause a synergistic effect on infectivity and/or transmission (Althaus et al., 2014). Despite this, research has focused on each pathogen individually, limiting our understanding of Ct/Ng co-infection and potential Ct/Ng interaction. Here, we developed an experimentally-tractable *in vitro* model of Ct/Ng co-infection, with specific focus on the effect Ng may exert on Ct inclusion development and infectivity.

To analyze CtE development/infectivity for the entire CtE developmental cycle, we required a system that prevented Ng-dependent host cell lysis. Near confluence and cycloheximide exposure allowed the host cell monolayer to survive for the duration of the experiments. Ng and Ct have both been previously reported to induce apoptosis and, in contrast, to inhibit induced-apoptosis (Gibellini et al., 1998; Müller et al., 1999; Müller et al., 2000; Schöier et al., 2001; Perfettini et al., 2002; Binnicker et al., 2003; Miyairi et al., 2006; Howie et al., 2008). Prevention of Ng-induced apoptosis may explain the protective effect of cycloheximide in our model. Cycloheximide does not negatively impact, and can even enhance, chlamydial growth/development (Ripa and Mardh, 1977) and does not negatively impact Ng growth (Brodeur et al., 1977), as we confirmed for the Ng strains used herein. Our model has the disadvantage of the less relevant setting of pharmacologically-limited host response to infection; though the resulting limited cell division might be somewhat representative of non-proliferative (e.g., senescent, terminally differentiated) host cells. However, because cycloheximide does not affect prokaryotic protein synthesis, the system nonetheless provides a unique platform for future analyses of bacterial gene expression and regulation as it relates to Ct/Ng co-infection.

Limited host protein synthesis does not preclude a role for the host cells in the observed Ng anti-chlamydial effects. Extracellular media pH reduction and/or gross nutrient depletion are not sufficient to explain our findings, suggesting frank competition for extracellular resources amongst host and pathogens is not a key factor. However, Ng may act *via* pre-existing intracellular host defense mechanisms such as cytokine or danger associated molecular pattern release, sequestration of intracellular resources or disruption of intracellular trafficking. Though many potential mechanisms remain to be investigated, we speculate that perturbation of intracellular iron or sphingomyelin may be particularly relevant. Iron is a limiting factor for both Ng and Ct growth (Serkin and Seifert, 2000; Vardhan et al., 2009), and iron deprivation is specifically associated with reduced Ct inclusion size and infectivity (Raulston, 1997). The host-derived lipid sphingomyelin, which can be converted from ceramide in Ct-infected cells (Elwell et al., 2011; as reviewed by Bastidas et al., 2013), is incorporated into chlamydial membranes and plays a role in both chlamydial replication (van Ooij et al., 2000; as reviewed by Elwell and Engel, 2012) and inclusion growth (Robertson et al., 2009; as reviewed by Elwell and Engel, 2012). Notably, Ng invasion of non-phagocytic human cell lines has been reported to activate sphingomyelinases and release ceramide from host cells (Grassmé et al., 1997).

Ng viability appears important for the observed anti-chlamydial effects, as they are abrogated in formalin- or gentamycin-killed Ng or Ng lysates. However, further evaluation is required to support this finding. Because we did not include measures of Ng copy numbers in our study, but only live Ng CFU to determine inoculum size, it remains possible that the live and dead Ng present in gentamycin-free experimental infections exceeded the numbers of dead Ng bacteria applied as killed Ng or lysates. However, because Ng exerted similar effects when added before or after CtE infection, it seems unlikely that Ng simply blockades CtE adherence/invasion at the host cell surface. Pronounced intimate association of Ng with both uninfected and CtE-infected HeLa may represent only Ng adherence or adherence and invasion. Ng invasion, as described for Ng in HeLa (Jarvis et al., 1999; Edwards et al., 2002), may be an important determinant of the observed anti-chlamydial effects and further work with adherence- and invasion-incompetent mutant Ng may help clarify this.

Preventing continuous Ng growth by adding gentamycin can abolish the effect of Ng on inclusion size, without abolishing the effect of Ng on chlamydial infectivity. We speculate that this effect may represent delayed developmental cycle progression or the induction of chlamydial persistence. We propose that: 1) anti-chlamydial effects of Ng on CtE inclusion formation and infectivity can occur, at least in part, independently of effects on inclusion size; and 2) Ng induced reduction of CtE inclusion size may depend on the duration and/or magnitude of active Ng growth during the CtE developmental cycle. Live Ng are required for the invasion of cultured epithelial cells (Bish et al., 2008) and we expected that gentamycin added concomitantly with Ng infection might allow limited Ng adherence/invasion but substantially limit the duration of Ng invasion of HeLa cells, ultimately preventing continued Ng growth in the culture medium of the model system.

We observed two instances of unexpected lack of reduced CtE infectivity despite reduced CtE inclusion size and/or numbers. First, Ng infection immediately after CtE infection (gentamycin-free experiment) reduced inclusion size and number, without a corresponding reduction in CtE infectivity. However, lowered media pH (i.e., from 7.4–6.5-7.0) in the absence of Ng infection, initiated after but not before CtE infection, increased infectivity 100%. Because Ng infection after CtE infection similarly reduces medium pH, we speculate that this pH-dependent increased CtE infectivity may effectively “mask” the reduced CtE titer expected to accompany reduced CtE inclusion size. This was reproduced with conditioned media generated from HeLa infected with Ng, which is also pH 6.5-7.0, and similarly causes a 100% increase in CtE infectivity when added after CtE infection. Intriguingly, conditioned media generated from CtE-infected HeLa subsequently infected with Ng (again pH 6.5-7.0) has no effect on CtE infectivity. This may indirectly suggest that the HeLa/CtE/Ng supernatant harbors a secreted factor only formed in the setting of co-infection, which may possess an anti-chlamydial effect “masked” by low pH. This possibility merits further investigation. Importantly, induced intracellular sphingomyelin depletion has been previously demonstrated to result in a similar unexpected lack of reduced chlamydial infectivity, despite marked reduced inclusion size (Hackstadt et al., 1996; Robertson et al., 2009). Specifically, incongruity between CtE inclusion size and infectivity/IFU upon sphingomyelin deficiency induced by myriocin treatment of HEp2 cells, (which inhibits the biosynthesis of sphingomyelin) resulted in very small inclusions with no appreciable reduction in infectivity (at 36 hpi), an effect associated with premature chlamydial differentiation to infectious EB (Robertson et al., 2009). Similarly, incongruity between C. trachomatis (L2) inclusion size and infectivity upon sphingomyelin deficiency induced by Brefeldin A treatment of HeLa 229 cells, (which disrupts “delivery” of host cell sphingomyelin to developing chlamydiae) resulted in small inclusions with no appreciable reduction in infectivity (Hackstadt et al., 1996). These two studies 1) strongly suggest that very small inclusions may feasibly yield unexpectedly high IFU/infectivity, and 2) further support our speculation that Ng-induced ceramide release may perturb intracellular sphingomyelin levels and represent a potential mechanism for the observed Ng-dependent anti-chlamydial effect on inclusion size.

Second, Ng infection immediately after CtE infection (gentamycin-containing experiment) reduced inclusion size and number without corresponding reduction in CtE infectivity. This could not be explained by decreased pH because gentamycin prevented pH reduction. However, gentamycin alone reduced CtE infectivity by 50%, potentially explaining the observed discrepancy. Gentamycin, though generally regarded as largely unable to penetrate host cells (Tabrizi and Robins-Browne, 1993; Marro et al., 2021), has been shown to enter host cells and affect the growth/development of intracellular bacteria (VanCleave et al., 2017). We suspect that the effect of gentamycin on CtE infectivity may have been ameliorated by Ng inoculum irreversibly binding gentamycin.

To our knowledge only two previous studies have focused on Ct/Ng co-infection, *in vitro* and/or *in vivo*. The first *C. muridarum* (Cm)/Ng co-infection model in mice was established in 2011 (Vonck et al., 2011). In this model, mice were vaginally infected first with Cm (to model human vaginal Ct infection) and then with Ng which increased Ng vaginal shedding in co-infected mice, compared to Ng-singly infected mice. This study provided the first experimental evidence in support of the clinical findings suggesting Ct/Ng synergy. A complementary *in vitro* experiment showed epithelial cells infected with *Chlamydia* did not increase Ng adherence or invasion, suggesting the Cm-dependent pro-gonorrheal effect observed *in vivo* was not due to increased Ng adherence or invasion (Vonck et al., 2011). Our observation of the intimate association of Ng, with both uninfected and CtE-infected HeLa,is congruent with the Vonck et al. *in vitro* findings.

Recently, Rajeeve et al. showed that the formation of anti-gonorrheal neutrophil extracellular traps (NETs) was prevented when PMNs were pre-infected with Ct (Rajeeve et al., 2018). Because anti-microbial NET formation also occurs in mice (Ermert et al., 2009; Rajeeve et al., 2018), the Rajeeve study provides a possible explanation for the *in vivo* pro-gonorrheal effect of Cm co-infection observed in the Vonck et al. study. The findings of these two previous studies, in general, suggest that Cm or Ct/Ng biological interaction may promote Ng infection and survival *in vivo*. Taken together, these two studies suggest that the pro-gonorrheal effect elicited by *Chlamydia* in the former study is more likely to operate *via* immune cells than the epithelial cells targeted directly by chlamydial or gonorrheal infection. This prediction is supported by the lack of a Ct/Ng synergistic effect we report here. However, incorporation of other factors, such as cytokines or isolated PMNs, associated with *in vivo* Ct and Ng single and co-infections into the *in vitro* model we present, may provide further insight.

In conclusion, we found no evidence, thus far, in support of our hypothesis that Ng-infected cells are more susceptible to CtE infection. However, our findings suggest: 1) Ng co-culture elicits an anti-chlamydial effect; 2) this anti-chlamydial effect can be associated with, or independent of, reduced chlamydial inclusion size; 3) Ng co-infection does not need to occur prior to chlamydial infection to elicit the observed anti-chlamydial effects; 4) the anti-chlamydial effect(s) appear to depend largely on Ng viability but are not mediated by media depletion of selected nutrients nor by Ng-dependent pH reduction; and 5) the factor(s) responsible for the Ng anti-chlamydial effect may be present in the conditioned culture medium. If so, further work is required to determine if the factor(s) originate from the Ng directly and/or from the host cell response to Ng and to identify the anti-chlamydial factors. These findings lead us to hypothesize that more than one mechanism/pathway likely plays a role in the observed Ng anti-chlamydial effects on inclusion size, inclusion number, and infectivity. Future studies will be needed to elucidate the mechanisms that lead to the anti-chlamydial effect of Ng as seen in the novel co-infection model we present here. Thus, we propose that this *in vitro* model will provide further insight into the pathogenic interplay between these two important bacteria.

## Data Availability Statement

The raw data supporting the conclusions of this article will be made available by the authors, without undue reservation.

## Author Contributions

NB and RS conceived the project, CL designed the experiments, DO and CL performed the experiments, DO and CL drafted the manuscript and all authors reviewed and edited the manuscript. All authors contributed to the article and approved the submitted version.

## Funding

This study was funded with funds from the Swiss National Science Foundation (https://www.snf.ch/en) under grant number 310030_179391 (NB). The funder had no role in study design, data collection/analysis or manuscript preparation.

## Conflict of Interest

The authors declare that the research was conducted in the absence of any commercial or financial relationships that could be construed as a potential conflict of interest.

## Publisher’s Note

All claims expressed in this article are solely those of the authors and do not necessarily represent those of their affiliated organizations, or those of the publisher, the editors and the reviewers. Any product that may be evaluated in this article, or claim that may be made by its manufacturer, is not guaranteed or endorsed by the publisher.

## References

[B1] AbdelRahman Y. M. Belland R. J. (2005). The Chlamydial Developmental Cycle. FEMS Microbiol. Rev. 29, 949–959. doi: 10.1016/j.femsre.2005.03.002 16043254

[B2] Abdelrahman Y. Ouellette S. P. Belland R. J. Cox J. V. Sassetti C. M. (2016). Polarized Cell Division of Chlamydia Trachomatis. PloS Pathog. 12 (8), e1005822. doi: 10.1371/journal.ppat.1005822 27505160 PMC4978491

[B3] Althaus C. L. Turner K. M. E. Mercer C. H. Auguste P. Roberts T. E. Bell G.. (2014). Effectiveness and Cost-Effectiveness of Traditional and New Partner Notification Technologies for Curable Sexually Transmitted Infections: Observational Study, Systematic Reviews and Mathematical Modelling. Health Technol. Assess. (Rockv). 18, 1–99. doi: 10.3310/hta18020 PMC4780998 24411488

[B4] Bastidas R. J. Elwell C. A. Engel J. N. Valdivia R. H. (2013). Chlamydial Intracellular Survival Strategies. Cold Spring Harb. Perspect. Med. 3 (5), a010256. doi: 10.1101/cshperspect.a010256 23637308 PMC3633179

[B5] Batteiger B. E. Fraiz J. Newhall V W. J. Katz B. P. Jones R. B. (1989). Association Of Recurrent Chlamydial Infection With Gonorrhea. J. Infect. Dis. 159, 661–669. doi: 10.1093/infdis/159.4.661 2926160

[B6] Binnicker J. Williams R. D. Apicella M. A. (2003). Infection of Human Urethral Epithelium With Neisseria Gonorrhoeae Elicits an Upregulation of Host Anti-Apoptotic Factors and Protects Cells From Staurosporine-Induced Apoptosis. Cell. Microbiol. 5, 549–560. doi: 10.1046/j.1462-5822.2003.00300.x 12864814

[B7] Bish S. E. Song W. Stein D. C. (2008). Quantification of Bacterial Internalization by Host Cells Using a B-Lactamase Reporter Strain: Neisseria Gonorrhoeae Invasion Into Cervical Epithelial Cells Requires Bacterial Viability. Microbes Infect. 10, 1182–1191. doi: 10.1016/j.micinf.2008.06.014 18678271 PMC2617741

[B8] Brodeur B. R. Johnson W. M. Johnson K. G. Diena B. B. (1977). *In Vitro* Interaction of Neisseria Gonorrhoeae Type 1 and Type 4 With Tissue Culture Cells. Infect. Immun. 15, 560–567. doi: 10.1128/IAI.15.2.560-567.1977 403139 PMC421404

[B9] Chao Y. Zhang T. (2011). Optimization of Fixation Methods for Observation of Bacterial Cell Morphology and Surface Ultrastructures by Atomic Force Microscopy. Appl. Microbiol. Biotechnol. 92, 381–392. doi: 10.1007/S00253-011-3551-5/TABLES/5 21881891 PMC3181414

[B10] Cohen M. S. Cannon J. G. Jerse A. E. Charniga L. M. Isbey S. F. Whicker L. G. (1994). Human Experimentation With Neisseria Gonorrhoeae: Rationale, Methods, and Implications for the Biology of Infection and Vaccine Development. J. Infect. Dis. 169, 532–537. doi: 10.1093/INFDIS/169.3.532 8158024

[B11] Creighton S. Tenant-Flowers M. Taylor C. B. Miller R. Low N. (2003). Co-Infection With Gonorrhoea and Chlamydia: How Much is There and What Does it Mean? Int. J. STD AIDS 14, 109–113. doi: 10.1258/095646203321156872 12662389

[B12] Department of Health and Human Services (2020). Sexually Transmitted Infections National Strategic Plan for the United States: 2021–2025. Dis. Control Div. (TB/Leprosy Sect., 12).

[B13] Edwards J. L. Brown E. J. Uk-Nham S. Cannon J. G. Blake M. S. Apicella M. A. (2002). A Co-Operative Interaction Between Neisseria Gonorrhoeae and Complement Receptor 3 Mediates Infection of Primary Cervical Epithelial Cells. Cell. Microbiol. 4, 571–584. doi: 10.1046/j.1462-5822.2002.t01-1-00215.x 12390350

[B14] Elwell C. A. Engel J. N. (2012). Lipid Acquisition by Intracellular Chlamydiae. Cell. Microbiol. 14, 1010–1018. doi: 10.1111/j.1462-5822.2012.01794.x 22452394 PMC3376245

[B15] Elwell C. A. Jiang S. Kim J. H. Lee A. Wittmann T. Hanada K.. (2011). Chlamydia Trachomatis Co-Opts Gbf1 and Cert to Acquire Host Sphingomyelin for Distinct Roles During Intracellular Development. PloS Pathog. 7 (9), e1002198. doi: 10.1371/JOURNAL.PPAT.1002198 21909260 PMC3164637

[B16] Elwell C. Mirrashidi K. Engel J. (2016). Chlamydia Cell Biology and Pathogenesis. Nat. Rev. Microbiol. 14, 385–400. doi: 10.1038/nrmicro.2016.30 27108705 PMC4886739

[B17] Ermert D. Urban C. F. Laube B. Goosmann C. Zychlinsky A. Brinkmann V. (2009). Mouse Neutrophil Extracellular Traps in Microbial Infections. J. Innate Immun. 1, 181–193. doi: 10.1159/000205281 20375576 PMC6951040

[B18] Gibellini D. Panaya R. Rumpianesi F. (1998). Induction of Apoptosis by Chlamydia Psittaci and Chlamydia Trachomatis Infection in Tissue Culture Cells. Zent. Bakteriol. 288, 35–43. doi: 10.1016/s0934-8840(98)80095-9 9728403

[B19] Grassmé H. Gulbins E. Brenner B. Ferlinz K. Sandhoff K. Harzer K.. (1997). Acidic Sphingomyelinase Mediates Entry of N. Gonorrhoeae Into Nonphagocytic Cells. Cell 91, 605–615. doi: 10.1016/S0092-8674(00)80448-1 9393854

[B20] Hackstadt T. Rockey D. D. Heinzen R. A. Scidmore M. A. (1996). Chlamydia Trachomatis Interrupts an Exocytic Pathway to Acquire Endogenously Synthesized Sphingomyelin in Transit From the Golgi Apparatus to the Plasma Membrane. EMBO J. 15 (5), 964–977. doi: 10.1002/j.1460-2075.1996.tb00433.x 8605892 PMC449991

[B21] Holmes K. K. Sparling P. F. Stamm W. E. Piot P. Wasserheit J. N. Corey L.. (2008). Sexually Transmitted Diseases. 4th ed (New York: McGraw Hill).

[B22] Holt S. Pedersen A. H. B. Wang S. P. Kenny G. E. Foy H. M. Grayston J. T. (1967). Isolation of Tric Agents and Mycoplasma From the Genito-Urinary Tracts of Patients of A Venereal Disease Clinic. Am. J. Ophthalmol. 63, 1057–1064. doi: 10.1016/0002-9394(67)94083-4 6025156

[B23] Howie H. L. Shiflett S. L. So M. (2008). Extracellular Signal-Regulated Kinase Activation by Neisseria Gonorrhoeae Downregulates Epithelial Cell Proapoptotic Proteins Bad and Bim. Infect. Immun. 76, 2715–2721. doi: 10.1128/IAI.00153-08 18391004 PMC2423055

[B24] Jarvis G. A. Li J. Swanson K. V. (1999). Invasion of Human Mucosal Epithelial Cells by Neisseria Gonorrhoeae Upregulates Expression of Intercellular Adhesion Molecule 1 (ICAM-1). Infect. Immun. 67, 1149–1156. doi: 10.1128/IAI.67.3.1149-1156.1999 10024555 PMC96441

[B25] Jerse A. E. Bash M. C. Russell M. W. Products A. (2015). Vaccines Against Gonorrhoea: Current Status and Future Challenges. Vaccine 32, 1579–1587. doi: 10.1016/j.vaccine.2013.08.067.Vaccines PMC4682887 24016806

[B26] Jones A. Jonsson A. Aro H. (2007). Neisseria Gonorrhoeae Infection Causes a G1 Arrest in Human Epithelial Cells. FASEB J. 21, 345–355. doi: 10.1096/FJ.06-6675COM 17158783

[B27] Leonard C. A. Schoborg R. V. Borel N. (2015). Damage/Danger Associated Molecular Patterns (DAMPs) Modulate Chlamydia Pecorum and C. Trachomatis Serovar E Inclusion Development *In Vitro* . PloS One 10, e0134943. doi: 10.1371/JOURNAL.PONE.0134943 26248286 PMC4527707

[B28] Leonard C. A. Schoborg R. V. Low N. Unemo M. Borel N. (2019). Pathogenic Interplay Between Chlamydia Trachomatis and Neisseria Gonorrhoeae That Influences Management and Control Efforts—More Questions Than Answers? Curr. Clin. Microbiol. Rep. 6, 182–191. doi: 10.1007/s40588-019-00125-4

[B29] Lin J. S. L. Donegan S. P. Heeren T. C. Greenberg M. Flaherty E. E. Haivanis R.. (1998). Transmission of Chlamydia Trachomatis and Neisseria Gonorrhoeae Among Men With Urethritis and Their Female Sex Partners. J. Infect. Dis. 178, 1707–1712. doi: 10.1086/314485 9815223

[B30] Lu P. Wang S. Lu Y. Neculai D. Sun Q. van der Veen S. (2019). A Subpopulation of Intracellular Neisseria Gonorrhoeae Escapes Autophagy-Mediated Killing Inside Epithelial Cells. J. Infect. Dis. 219, 133–144. doi: 10.1093/infdis/jiy237 29688440

[B31] Marangoni A. Bergamini C. Fato R. Cavallini C. Donati M. Nardini P.. (2014). Infection of Human Monocytes by Chlamydia Pneumoniae and Chlamydia Trachomatis: An *In Vitro* Comparative Study. BMC Res. Notes 7, 1–9. doi: 10.1186/1756-0500-7-230/FIGURES/6 24721461 PMC3984436

[B32] Markle W. Conti T. Kad M. (2013). Sexually Transmitted Diseases. Prim. Care - Clin. Off. Pract. 40, 557–587. doi: 10.1016/j.pop.2013.05.001 23958358

[B33] Marro F. C. Abad L. Blocker A. J. Laurent F. Josse J. Valour F. (2021). *In Vitro* Antibiotic Activity Against Intraosteoblastic Staphylococcus Aureus: A Narrative Review of the Literature. J. Antimicrob. Chemother. 76, 3091–3102. doi: 10.1093/jac/dkab301 34459881 PMC8598303

[B34] Medline A. Joseph Davey D. Klausner J. D. (2017). Lost Opportunity to Save Newborn Lives: Variable National Antenatal Screening Policies for Neisseria Gonorrhoeae and Chlamydia Trachomatis. Int. J. STD AIDS 28, 660–666. doi: 10.1177/0956462416660483 27440873 PMC6879101

[B35] Moore E. R. Fischer E. R. Mead D. J. Hackstadt T. (2008). The Chlamydial Inclusion Preferentially Intercepts Basolaterally Directed Sphingomyelin-Containing Exocytic Vacuoles. Traffic 9 (12), 2130–40 2130. doi: 10.1111/J.1600-0854.2008.00828.X 18778406 PMC2951019

[B36] Miyairi I. Byrne G. I. (2006). Chlamydia and Programmed Cell Death. Curr Opin Microbiol. 9 (1), 102–108. doi: 10.1016/j.mib.2005.12.004 16406838

[B37] Müller A. Günther D. Brinkmann V. Hurwitz R. Meyer T. F. Rudel T. (2000). Targeting of the Pro-Apoptotic VDAC-Like Porin (PorB) of Neisseria Gonorrhoeae to Mitochondria of Infected Cells. EMBO J. 19, 5332–5343. doi: 10.1093/EMBOJ/19.20.5332 11032801 PMC314008

[B38] Müller A. Günther D. Düx F. Naumann M. Meyer T. F. Rudel T. (1999). Neisserial Porin (PorB) Causes Rapid Calcium Influx in Target Cells and Induces Apoptosis by the Activation of Cysteine Proteases. EMBO J. 18, 339–352. doi: 10.1093/EMBOJ/18.2.339 9889191 PMC1171129

[B39] Onorini D. Donati M. Marti H. Biondi R. Levi A. Nufer L.. (2019). The Influence of Centrifugation and Incubation Temperatures on Various Veterinary and Human Chlamydial Species. Vet. Microbiol. 233, 11–20. doi: 10.1016/j.vetmic.2019.04.012 31176395

[B40] Ouellette S. P. Lee J. Cox J. V. (2020). Division Without Binary Fission: Cell Division in the FtsZ-Less Chlamydia. J. Bacteriol. 202 (17), e00252–e00220. doi: 10.1128/JB.00252-20 32540934 PMC7417837

[B41] Patrone J. B. Bish S. E. Stein D. C. (2006). TNF-α-Independent IL-8 Expression: Alterations in Bacterial Challenge Dose Cause Differential Human Monocytic Cytokine Response. J. Immunol. 177, 1314–1322. doi: 10.4049/JIMMUNOL.177.2.1314 16818792

[B42] Perfettini J.-L. Reed J. C. Israël N. Martinou J.-C. Dautry-Varsat A. Ojcius D. M. (2002). Role of Bcl-2 Family Members in Caspase-Independent Apoptosis During Chlamydia Infection. Infect. Immun. 70, 55–61. doi: 10.1128/IAI.70.1.55-61.2002 11748163 PMC127616

[B43] Pettengill M. A. Marques-da-Silva C. Avila M. L. Oliveira S. d. A. dos S. Lam V. W.. (2012). Reversible Inhibition of Chlamydia Trachomatis Infection in Epithelial Cells Due to Stimulation of P2X4 Receptors. Infect. Immun. 80, 4232. doi: 10.1128/IAI.00441-12 22988022 PMC3497399

[B44] Pollard D. A. Pollard T. D. Pollard K. S. (2019). Empowering Statistical Methods for Cellular and Molecular Biologists. Mol. Biol. Cell 30, 1359–1368. doi: 10.1091/mbc.E15-02-0076 31145670 PMC6724699

[B45] Quillin S. J. Seifert H. S. (2018). Neisseria Gonorrhoeae Host Adaptation and Pathogenesis. Nat. Rev. Microbiol. 16, 226–240. doi: 10.1038/nrmicro.2017.169 29430011 PMC6329377

[B46] Rajeeve K. Das S. Prusty B. K. Rudel T. (2018). Chlamydia Trachomatis Paralyses Neutrophils to Evade the Host Innate Immune Response. Nat. Microbiol. 3, 824–835. doi: 10.1128/JB.00252-20 29946164

[B47] Raulston J. E. (1997). Response of Chlamydia Trachomatis Serovar E to Iron Restriction *In Vitro* and Evidence for Iron-Regulated Chlamydial Proteins. Infect. Immun. 65, 4539–4547. doi: 10.1128/iai.65.11.4539-4547.1997 9353031 PMC175652

[B48] R Core Team (2020). R: A Language and Environment for Statistical Computing (Vienna, Austria: R Foundation for Statistical Computing). Available at: https://www.R-project.org/.

[B49] Richmond S. J. Hilton A. L. Clarke S. K. (1972). Chlamydial Infection. Role of Chlamydia Subgroup A in non-Gonococcal and Post-Gonococcal Urethritis. Br. J. Vener. Dis. 48, 437–444. doi: 10.1136/STI.48.6.437 4651178 PMC1048364

[B50] Ripa K. T. Mardh A. (1977). Cultivation of Chlamydia Trachomatis in Cycloheximide-Treated McCoy Cells. J. Clin. Microbiol. 6, 328–331. doi: 10.1128/jcm.6.4.328-331.1977 562356 PMC274768

[B51] Robertson D. K. Gu L. Rowe R. K. Beatty W. L. (2009). Inclusion Biogenesis and Reactivation of Persistent Chlamydia Trachomatis Requires Host Cell Sphingolipid Biosynthesis. PloS Pathog. 5 (11), e1000664. doi: 10.1371/JOURNAL.PPAT.1000664 19936056 PMC2774160

[B52] Rowley J. Hoorn S. V. Korenromp E. Low N. Unemo M. Abu-Raddad L. J.. (2019). Chlamydia, Gonorrhoea, Trichomoniasis and Syphilis. Bull. World Health Organ. 97, 548–562. doi: 10.2471/BLT.18.228486 31384073 PMC6653813

[B53] Schöier J. Ollinger K. Kvarnström M. Söderlund G. Kihlström E. (2001). Chlamydia Trachomatis-Induced Apoptosis Occurs in Uninfected McCoy Cells Late in the Developmental Cycle and is Regulated by the Intracellular Redox State. Microb. Pathog. 31 (4), 173–184. doi: 10.1006/mpat.2001.0460 11562170

[B54] Serkin C. D. Seifert H. S. (2000). Iron Availability Regulates DNA Recombination in Neisseria Gonorrhoeae. Mol. Microbiol. 37, 1075–1086. doi: 10.1046/j.1365-2958.2000.02058.x 10972826

[B55] Spurbeck R. R. Arvidson C. G. (2008). Inhibition of Neisseria Gonorrhoeae Epithelial Cell Interactions by Vaginal Lactobacillus Species. Infect. Immun. 76, 3124–3130. doi: 10.1128/IAI.00101-08 18411284 PMC2446695

[B56] Stupiansky N. W. van der Pol B. Williams J. A. Weaver B. Taylor S. E. Fortenberry J. D. (2011). The Natural History of Incident Gonococcal Infection in Adolescent Women. Sex Transm. Dis. 38, 750–754. doi: 10.1097/OLQ.0b013e31820ff9a4 21317686

[B57] Tabrizi S. N. Robins-Browne R. M. (1993). Elimination of Extracellular Bacteria by Antibiotics in Quantitative Assays of Bacterial Ingestion and Killing by Phagocytes. J. lmmunol. Methods 158 (2), 201–206. doi: 10.1016/0022-1759(93)90215-s 8429226

[B58] Unemo M. Shafer W. M. (2014). Antimicrobial Resistance in Neisseria Gonorrhoeae in the 21st Century: Past, Evolution, and Future. Clin. Microbiol. Rev. 27 (3), 587–613. doi: 10.1128/CMR.00010-14 24982323 PMC4135894

[B59] VanCleave T. T. Pulsifer A. R. Connor M. G. Warawa J. M. Lawrenz M. B. (2017). Impact of Gentamicin Concentration and Exposure Time on Intracellular Yersinia Pestis. Front. Cell. Infect. Microbiol. 7. doi: 10.3389/fcimb.2017.00505 PMC5732358 29312891

[B60] van Ooij C. Kalman L. Van Ijzendoorn S. Nishijima M. Hanada K. Mostov K.. (2000). Host Cell-Derived Sphingolipids are Required for the Intracellular Growth of Chlamydia Trachomatis. Cell. Microbiol. 2, 627–637. doi: 10.1046/J.1462-5822.2000.00077.X 11207614

[B61] Vardhan H. Bhengraj A. R. Jha R. Singh Mittal A. (2009). Chlamydia Trachomatis Alters Iron-Regulatory Protein-1 Binding Capacity and Modulates Cellular Iron Homeostasis in HeLa-229 Cells. J. Biomed. Biotechnol. 7, 342032. doi: 10.1155/2009/342032 PMC2727623 19688112

[B62] Vonck R. A. Darville T. O’Connell C. M. Jerse A. E. (2011). Chlamydial Infection Increases Gonococcal Colonization in a Novel Murine Coinfection Model. Infect. Immun. 79, 1566–1577. doi: 10.1128/IAI.01155-10 21245268 PMC3067530

[B63] World Health Organization (WHO) (2018) Report on global sexually transmitted infection surveillance. Available at: https://www.who.int/reproductivehealth/publications/stis-surveillance-2018/en/.

