# Supplementary material for: Neisseria gonorrhoeae Limits Chlamydia trachomatis Inclusion Development and Infectivity in a Novel In Vitro Co-Infection Model
Source: Front Cell Infect Microbiol. 2022 Jul 7;12:911818. doi: 10.3389/fcimb.2022.911818 (PMC9300984; doi:10.3389/fcimb.2022.911818)
Supplement: Supplementary file 1 [file DataSheet_1.pdf]

Supplementary Text 1 - Supplementary Materials and Methods

1. Mycoplasma testing

According to a previous study (Sigar et al., 2018) cell culture as well as chlamydial stocks are commonly contaminated with *Mycoplasma* spp. Therefore, DNA extracted from cells and chlamydial stocks were tested for mycoplasma contamination, using the Mycoplasma Detection Kit for conventional PCR per manufacturer's instructions (Venor®GeM OneStep, MB Minerva Biolabs, Germany). All cells and chlamydial stocks used in this study were *Mycoplasma* negative.

2. Determination of minimum Ng inocula required for growth in culture medium

Ng grown on chocolate agar for 18-24 h after sub-passage was suspended and serially diluted in 200 µl/well of culture medium in 96 well plates, with 8 replicate wells per inoculum. Plates were incubated at 37°C, 5% CO_2. Bacterial growth was visible by standard light microscopy after 24 h, visible by eye as turbidity after 48 h, and maximal growth was visible as turbid, acidified medium (as indicated by yellow color of phenol red indicator in medium) by 72 h. The following inocula, referred to herein as "minimum" Ng inoculum (PPNG 10^5 CFU/ml, FA 10^4 CFU/ml and NG 10^1 CFU/ml), represent 10^1 higher than the minimum inocula that resulted in growth in all 8 replicate wells for each strain.

3. Trypan blue assay

For analysis of cell membrane permeabilization, standard trypan blue exclusion analysis was modified for use on intact cell monolayer essentially as previously described (Perry et al., 1997; Çelik-Uzuner, 2018). Briefly, at the experimental endpoint, cells were washed three times with PBS, incubated with undiluted trypan blue reagent for 3 minutes, fixed with 4% paraformaldehyde for 30 minutes, dipped in PBS to rinse. Coverslips mounted on glass slides using FluoreGuard mounting medium (Hard Set; ScyTek Laboratories Inc.) were evaluated using the transmitted light setting of a Leica DMLB fluorescence microscope (Leica Microsystems, Wetzlar, Germany). To determine the number of permeabilized (blue) cells/field, 30 randomly selected reticle-delimited fields per coverslip were examined at 1000X (oil) magnification.

4. Determination of the effect of pH on *C. trachomatis* infection

The reduction of pH caused by Ng growth in our model was estimated by comparison of phenol red indicator color in the experimental culture medium to that of a self-prepared reference range of pH-adjusted cycloheximide-supplemented culture media. The range comprised pH 7.4 (normal culture medium, no pH adjustment), pH 7.0, pH 6.5, and pH 6.0. The effect of reduced pH alone on CtE infection was determined by exposure of CtE-infected HeLa to the pH-adjusted cycloheximide-supplemented culture media as per the two experimental co-infection settings, pre-infection of CtE with Ng (**Figure 1A**) and infection with Ng immediately after CtE infection (**Figure 1B**). To simulate Ng pre-infection, HeLa cells were exposed to the pH-adjusted cycloheximide-supplemented culture media for 24 h prior to CtE infection; immediately after CtE infection inocula were replaced with fresh pH-adjusted cycloheximide-supplemented culture media. To simulate Ng infection after CtE infection, HeLa cells were exposed to normal cycloheximide-supplemented culture medium (pH 7.4) for 24 h prior to subsequent CtE infection; immediately after CtE infection, inocula were replaced with fresh pH-adjusted cycloheximide-supplemented culture media. In both experimental settings, pH-adjusted cycloheximide-supplemented culture media was refreshed 20 h after CtE infection and samples were

processed for IF analysis of inclusion development/formation or determination of infectivity (IFU/well) at 40 h post-CtE infection.

5. Culture medium nutrient supplementation

To determine the effect of culture medium nutrient supplementation on Ng anti-chlamydial effects, HeLa cells were infected with 10^4 CFU/ml of FA suspended in culture medium supplemented with cycloheximide alone, or with cycloheximide and additional supplements (2X glucose, 5X GlutaMAX, 5X essential amino acids (AA) and 5X MEM NEAA, or a combination of 2X glucose, 5X GlutaMAX, 5X AA and MEM NEAA) for 24 h prior to subsequent CtE infection. Immediately after CtE infection, inocula were replaced with nutrient-supplemented cycloheximide-supplemented culture media and after 20 more h, these supplemented media were refreshed. Samples were processed for IF analysis of inclusion development/formation or determination of infectivity (IFU/well) at 40 h post-CtE infection.

6. CtE/Ng sequential immunostaining; immunofluorescence (IF) microscopy

At experimental endpoints, culture medium was removed from infected cells, cells were fixed with ice cold absolute methanol for 10 minutes and methanol was replaced with sterile PBS. First, chlamydial inclusions were immunostained with: 1:400-diluted *Chlamydiaceae* family-specific mouse monoclonal chlamydial anti-lipopolysaccharide(LPS) antibody (Clone ACI-P, Progen, Heidelberg, Germany) and 1:750-diluted Alexa Fluor 488-conjugated secondary goat anti-mouse antibody (Alexa 488; Life Technologies, Thermo Fisher Scientific). Then Ng was immunostained with: 1:1000-diluted rabbit polyclonal anti-Ng antibody (PAB29800, Abnova, Taipei City, Taiwan) and 1:750-diluted Alexa Fluor 488-conjugated secondary goat anti-mouse antibody (Alexa 594; Life Technologies, Thermo Fisher Scientific). Finally, DNA was stained with 1 µg/ml 4’, 6-diamidino-2’-phenylindole dihydrochloride (DAPI); Molecular Probes, Thermo Fisher Scientific). Coverslips mounted on glass slides using FluoreGuard mounting medium (Hard Set; ScyTek Laboratories Inc.) were evaluated using a Leica DMLB fluorescence microscope (Leica Microsystems, Wetzlar, Germany). Micrographs were generated using BonTec software (BonTec, Bonn, Germany) and a UI-2250SE-C-HQ camera (uEye, IDS Imaging Development Systems GmbH, Obersulm, Germany).

7. Generation of gentamycin- and formalin-killed FA and FA lysate; CtE-infected HeLa exposure

To generate killed FA, FA grown on chocolate agar for 18-24 h after sub-passage was suspended (10^8 CFU/ml) in cycloheximide-supplemented culture medium with either 50 µg/ml gentamycin or 200 µg/ml gentamycin at 37°C for 2 hours, or in PBS with 4% formalin at room temperature for 30 minutes. The FA was then pelleted and washed in PBS twice before being resuspended in cycloheximide-supplemented culture medium, again to 10^8 CFU/ml. HeLa cells were exposed to 2 ml/well resuspended killed FA. To generate FA lysate, FA grown on chocolate agar was suspended (10^8 CFU/ml) in PBS and frozen at -20°C. The frozen FA suspension was thawed, vortexed and re-frozen again a second time. The twice-thawed FA suspension was vortexed, centrifuged at 4°C for 30 minutes at 4122 g, and the cleared lysate was filter sterilized (0.22 µm). HeLa cells were exposed to 200 µl/well FA lysate, generated from 10^6 CFU, (or 200 µl PBS alone as a control) in cycloheximide-supplemented culture media (2 ml total volume per well). Before use in experiments, the resuspended FA and FA lysate were plated to chocolate agar to confirm lack of viable FA.

The killed FA and FA lysate were evaluated in both experimental settings (**Figures 1A, B**). To simulate Ng pre-infection, HeLa cells were exposed to killed FA or FA lysate in cycloheximide-supplemented culture medium for 24 h prior to subsequent CtE infection; immediately after CtE infection inocula

were replaced with killed FA or FA lysate cycloheximide-supplemented culture medium. To simulate Ng infection after CtE, HeLa cells were exposed cycloheximide-supplemented culture medium (no killed FA or FA lysate) for 24 h prior to subsequent CtE infection; immediately after CtE infection, inocula were replaced with killed FA or FA lysate in cycloheximide-supplemented culture medium. In both experimental settings, killed FA or FA lysate in cycloheximide-supplemented culture medium was refreshed 20 h after CtE infection and samples were processed for IF analysis of inclusion development/formation or determination of infectivity (IFU/well) at 40 h post-CtE infection.

8. Generation of conditioned media; CtE-infected HeLa exposure

Various conditioned media (cm) were generated to determine if anti-chlamydial factors associated with CtE/Ng co-infection might be secreted into the culture medium: HeLa cm, FA cm, HeLa/FA cm, HeLa/CtE cm and HeLa/CtE/FA cm. FA (10^6 CFU/well) and CtE (MOI 0.225) were used to infect HeLa in replicate wells in 24 well plates (2 ml final volume medium per well). For HeLa cm, mock CtE infection of HeLa cells (inoculation with culture medium only) was followed by replacement of inoculum with cycloheximide-supplemented culture medium; for FA cm, wells with no cells were prepared with FA in cycloheximide-supplemented culture medium; for HeLa/CtE cm, CtE infection of HeLa cells was followed by replacement of inoculum with cycloheximide-supplemented culture medium; for HeLa/CtE/FA cm, CtE infection of HeLa cells was followed by replacement of inoculum with 10^6 CFU FA in cycloheximide-supplemented culture medium. Plates were incubated at 37°C, 5% CO_2 for 24 h. The cm were pooled into separate 50 ml tubes and centrifuged at 4122 g for 30 minutes at 4C, followed by filtration of the cleared supernatants with sterile 0.22 µm filters. The cleared and filtered cm were plated to chocolate agar to confirm lack of viable FA. HeLa cells were exposed to 2 ml/well cm.

The cm were evaluated in both experimental settings (Figures 1A, B). To simulate Ng pre-infection, HeLa cells were exposed to cm for 24 h prior to subsequent CtE infection and immediately after CtE infection, inocula were replaced with cm. To simulate Ng infection after CtE, HeLa cells were exposed cycloheximide-supplemented culture medium for 24 h prior to subsequent CtE infection and cm were added immediately after CtE infection. In both experimental settings, cm was refreshed 20 h after CtE infection and samples were processed for IF analysis of inclusion development/formation or determination of infectivity (IFU/well) at 40 h post-CtE infection.

Supplementary Materials and Methods References

Çelik-Uzuner, S. (2018). Development of a Direct Trypan Blue Exclusion Method to Detect Cell Viability of Adherent Cells into ELISA Plates. Celal Bayar Üniversitesi Fen Bilim Derg 14: 99–104. doi:10.18466/cbayarfbe.372192.

Perry, S.W, Epstein, L.G., Gelbard, H.A. (1997). In Situ Trypan Blue Staining of Monolayer Cell Cultures for Permanent Fixation and Mounting. Biotechniques 22: 1020–1024. doi:10.2144/97226bm01.

Sigar, I.M., Schripsema, J.H., Kelly, K.A., Murthy, A.K., Manam, S., Ramsey, K.H. (2018). Elimination of Mycoplasma contamination in Chlamydia stocks as a result of in vivo passage or plaque isolation. BMC Res Notes. BioMed Central 11: 1–5. doi:10.1186/s13104-018-3455-x.
