# Supplementary figures and images for: Neisseria gonorrhoeae Limits Chlamydia trachomatis Inclusion Development and Infectivity in a Novel In Vitro Co-Infection Model
Source: Front Cell Infect Microbiol. 2022 Jul 7;12:911818. doi: 10.3389/fcimb.2022.911818 (PMC9300984; doi:10.3389/fcimb.2022.911818)

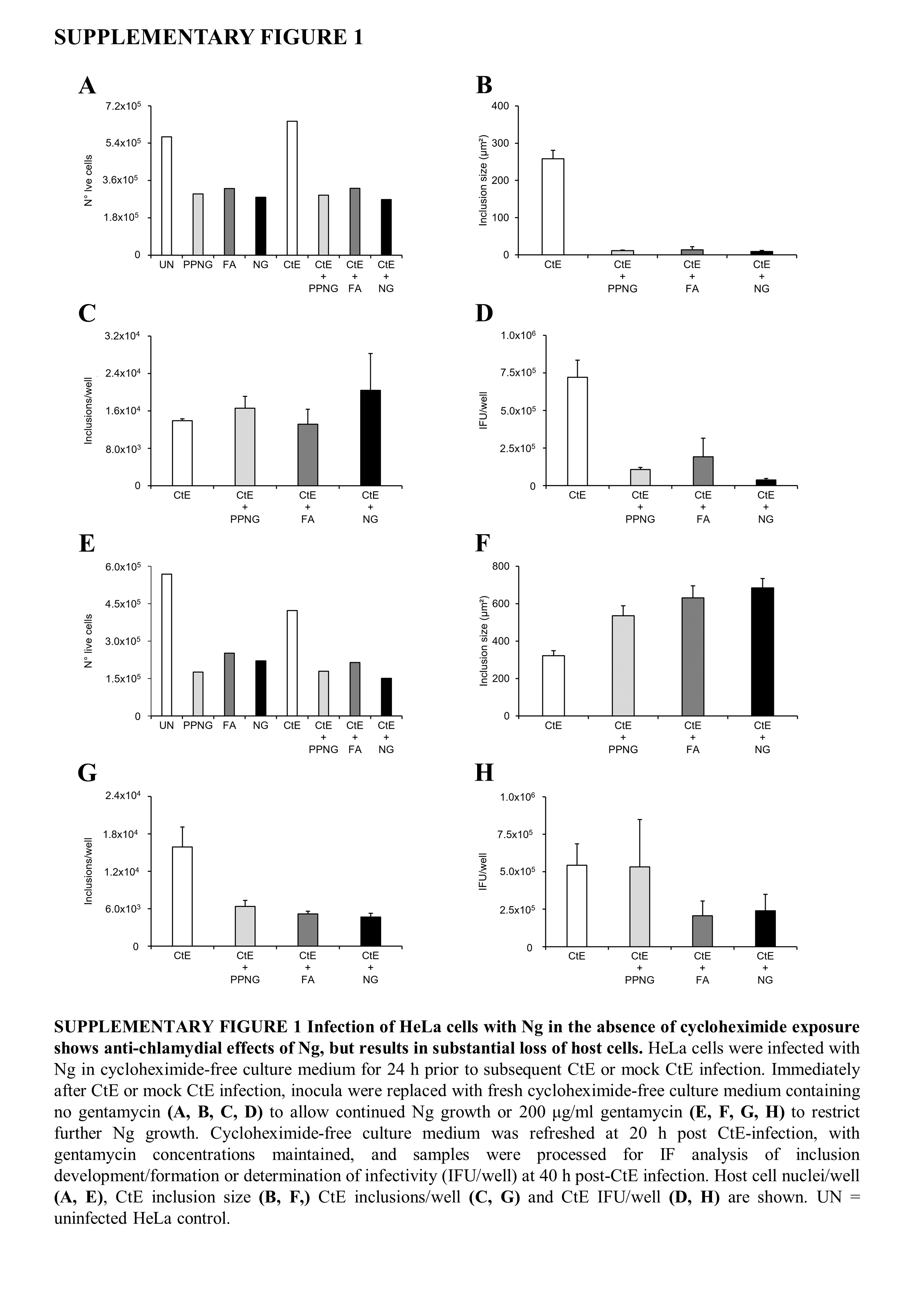

Supplement: Supplementary file 3 [file Image_1.tif]

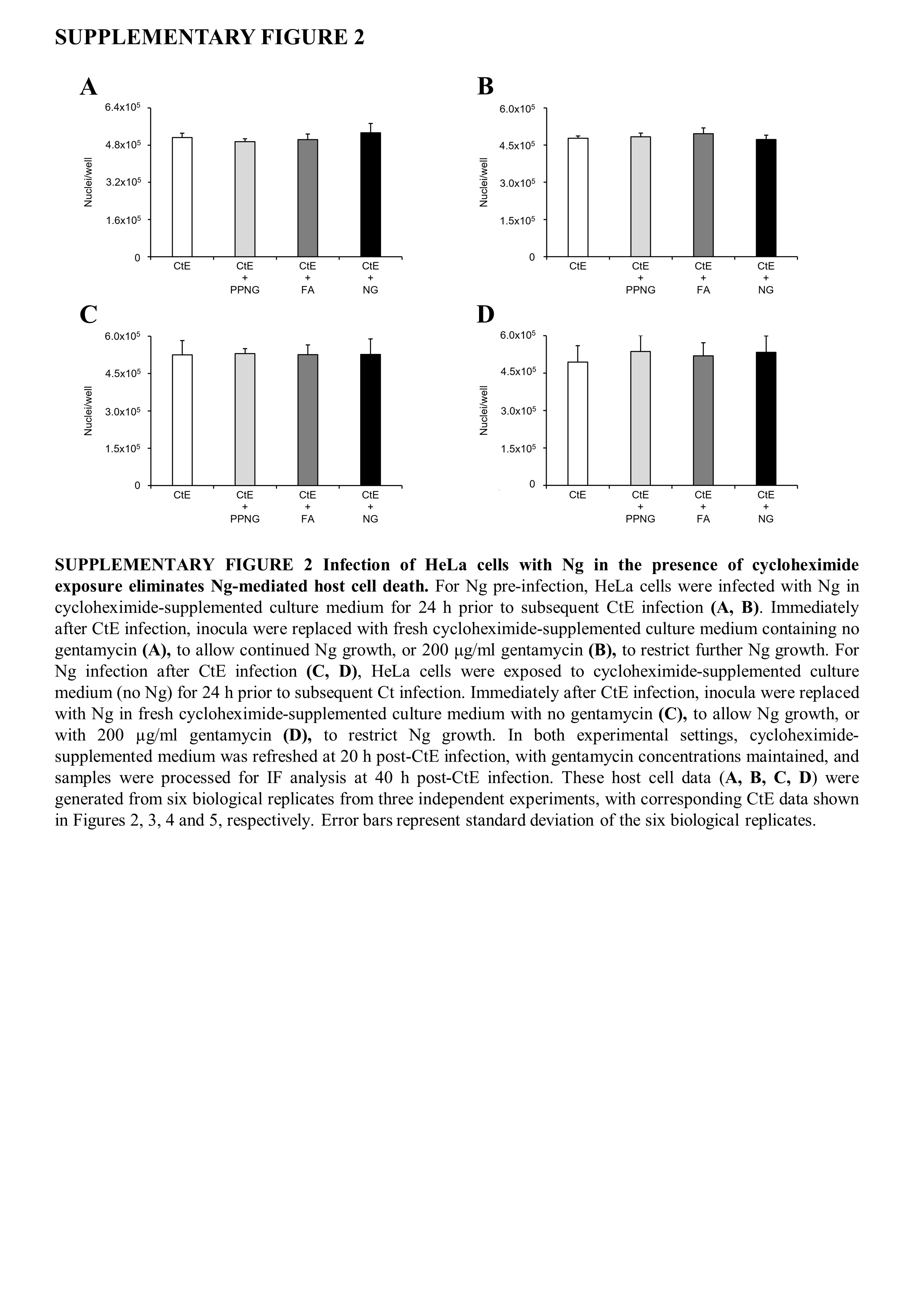

Supplement: Supplementary file 4 [file Image_2.tif]

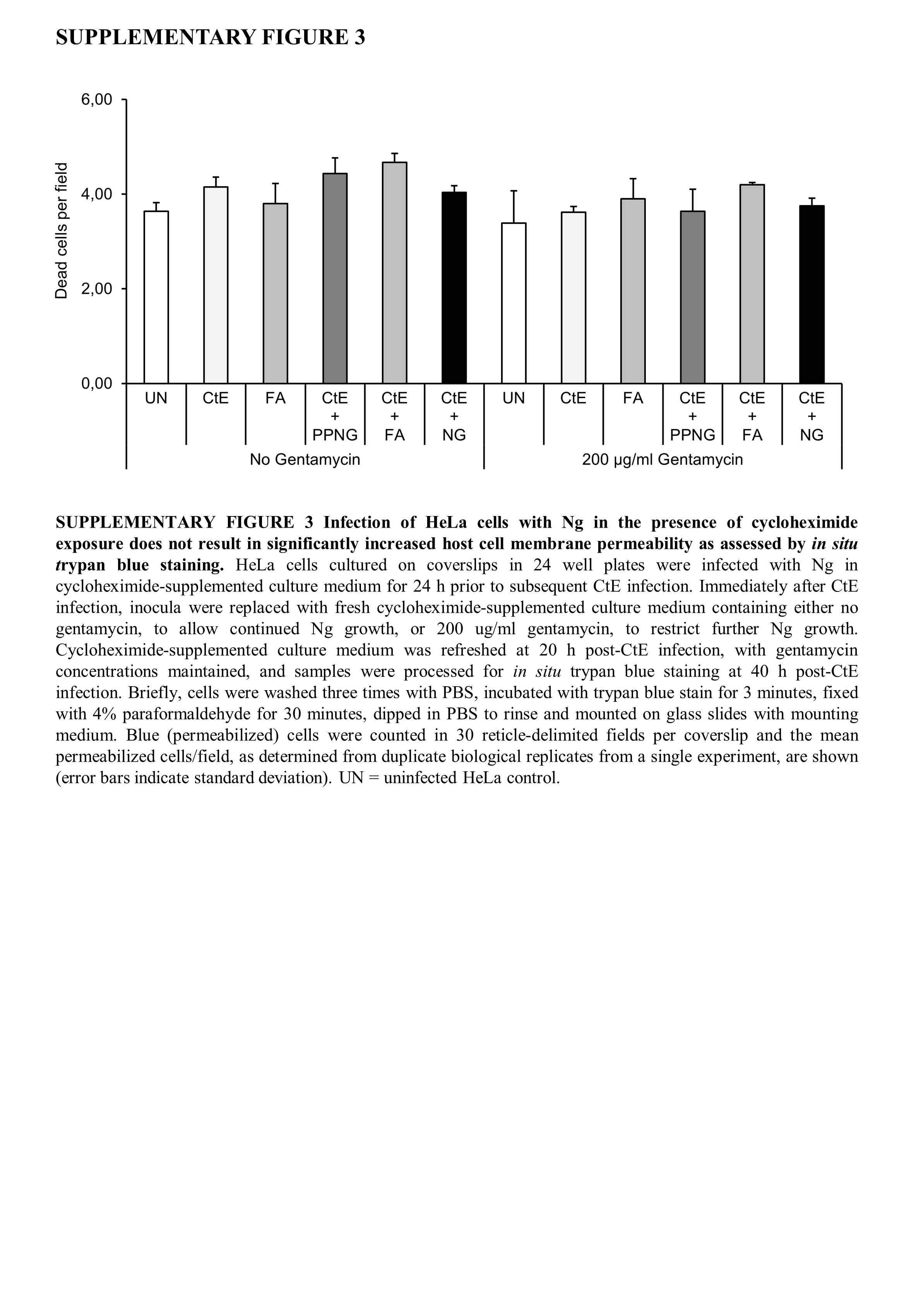

Supplement: Supplementary file 5 [file Image_3.tif]

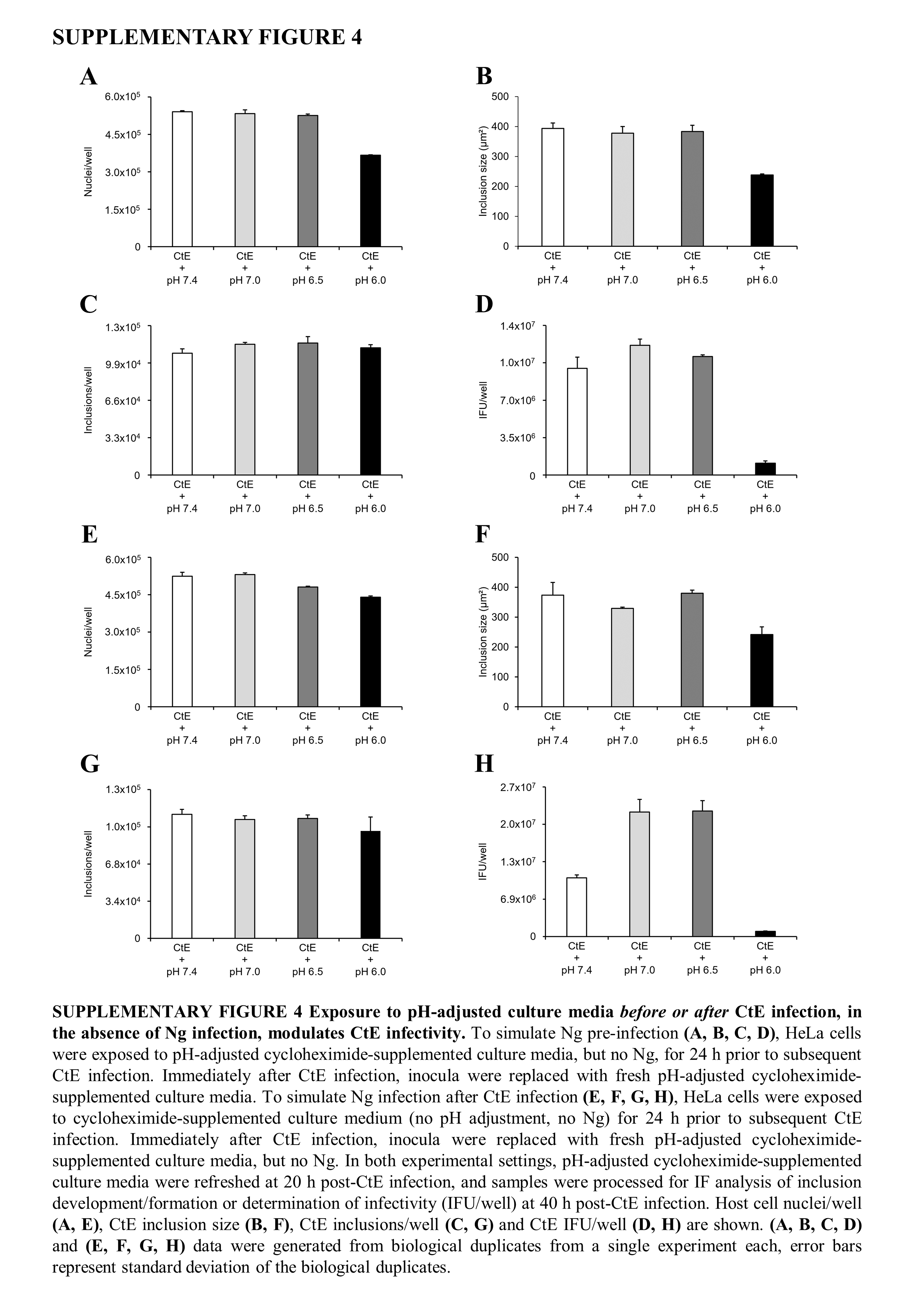

Supplement: Supplementary file 6 [file Image_4.tif]

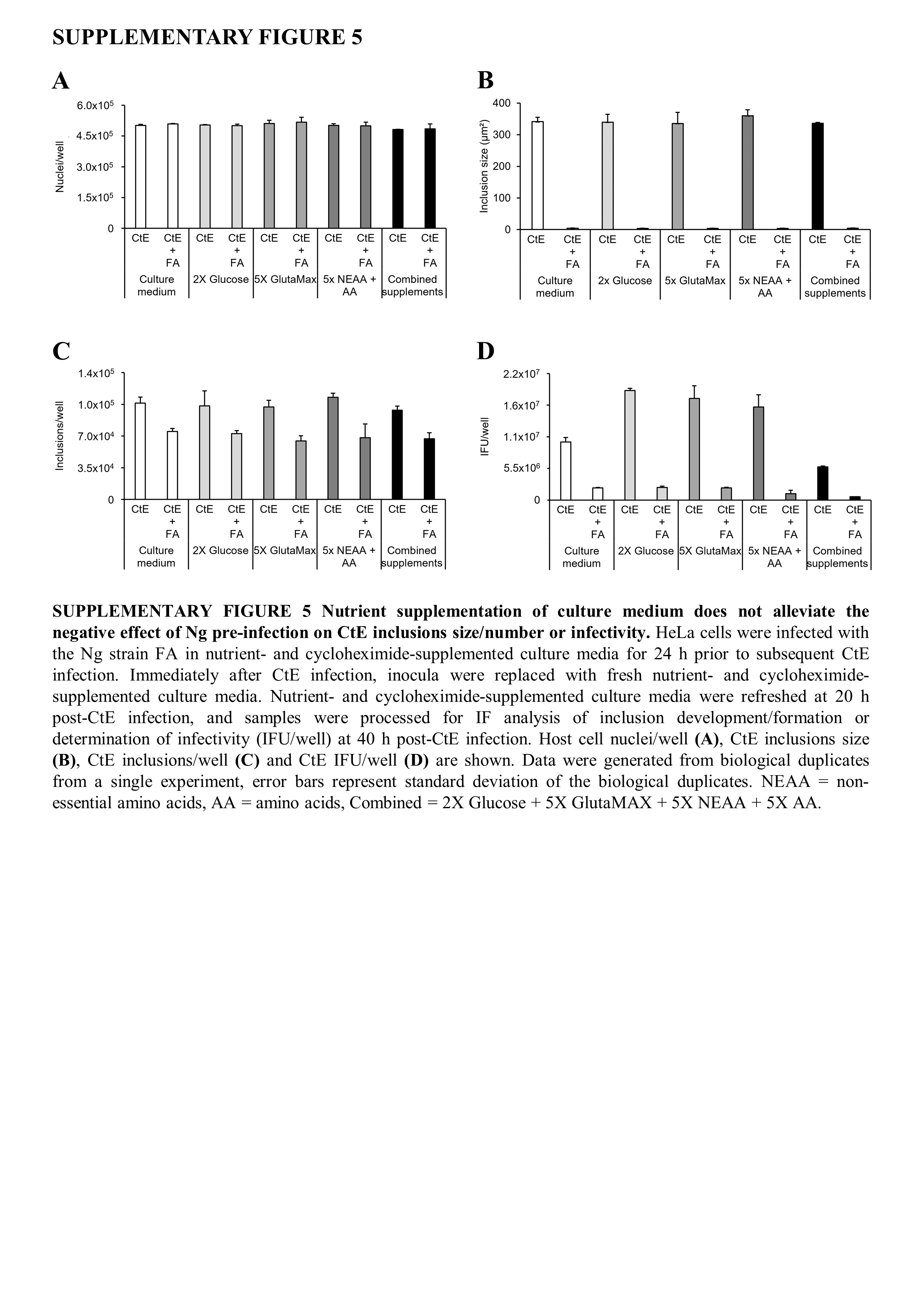

Supplement: Supplementary file 7 [file Image_5.tif]

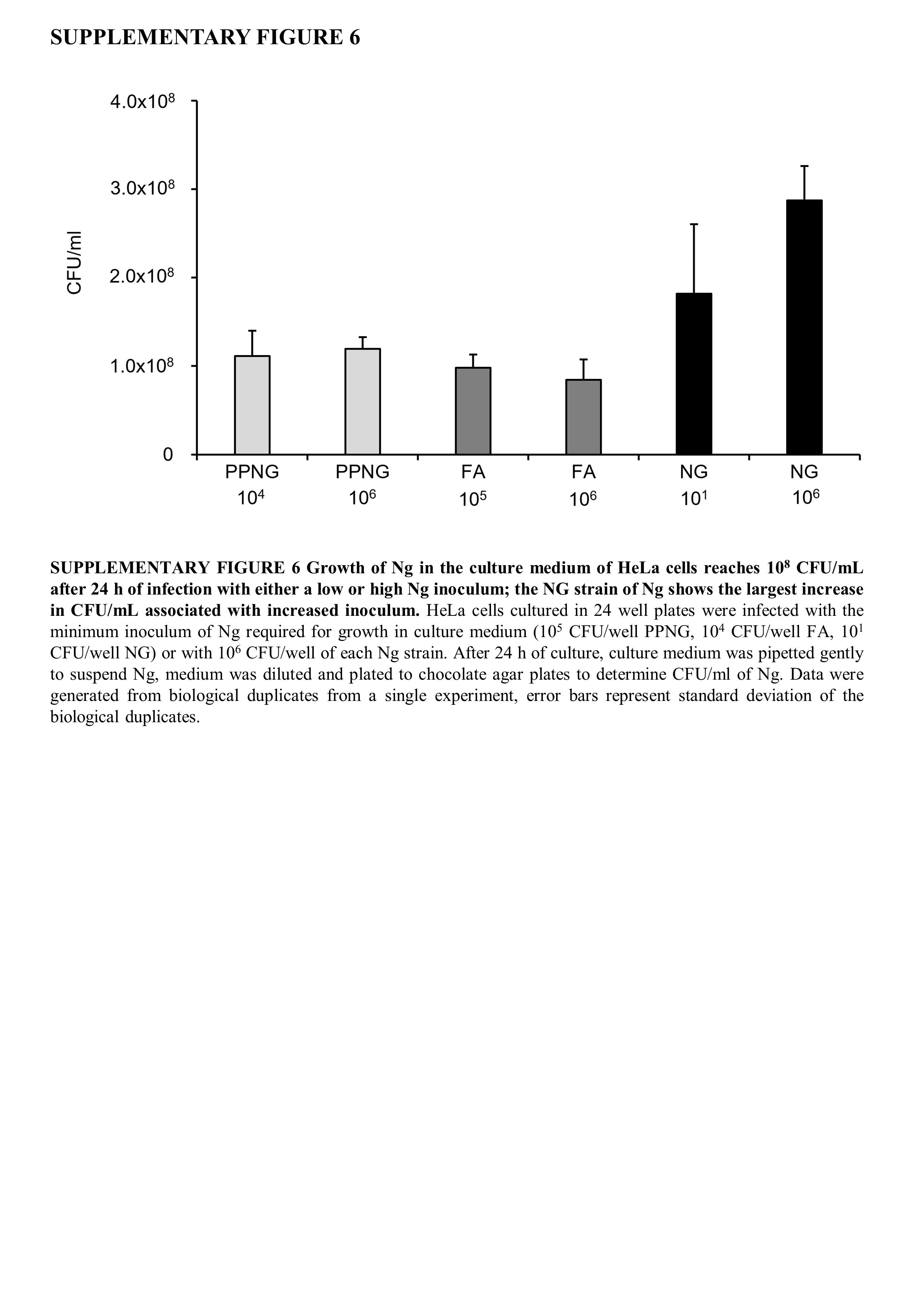

Supplement: Supplementary file 8 [file Image_6.tif]

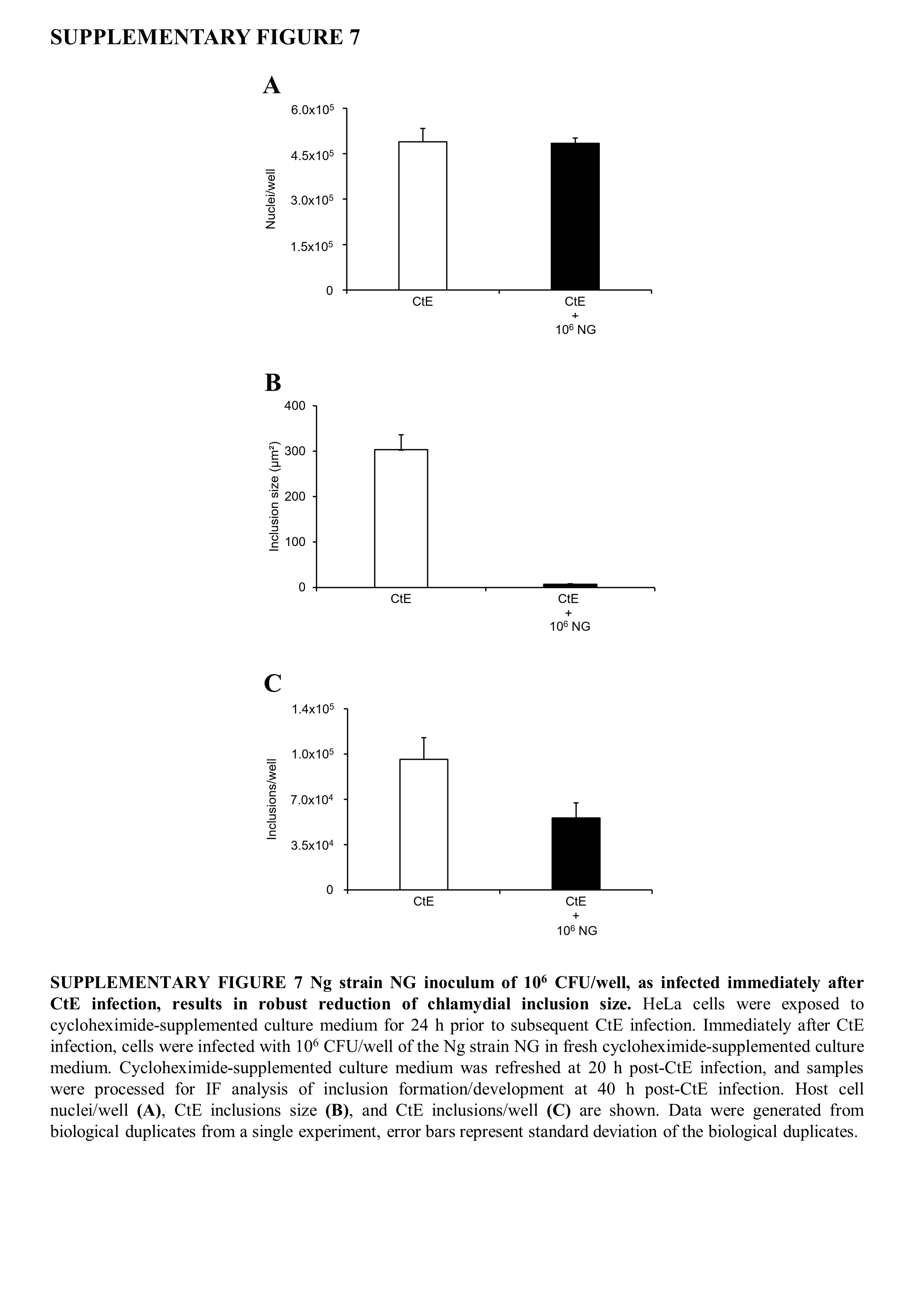

Supplement: Supplementary file 9 [file Image_7.tif]

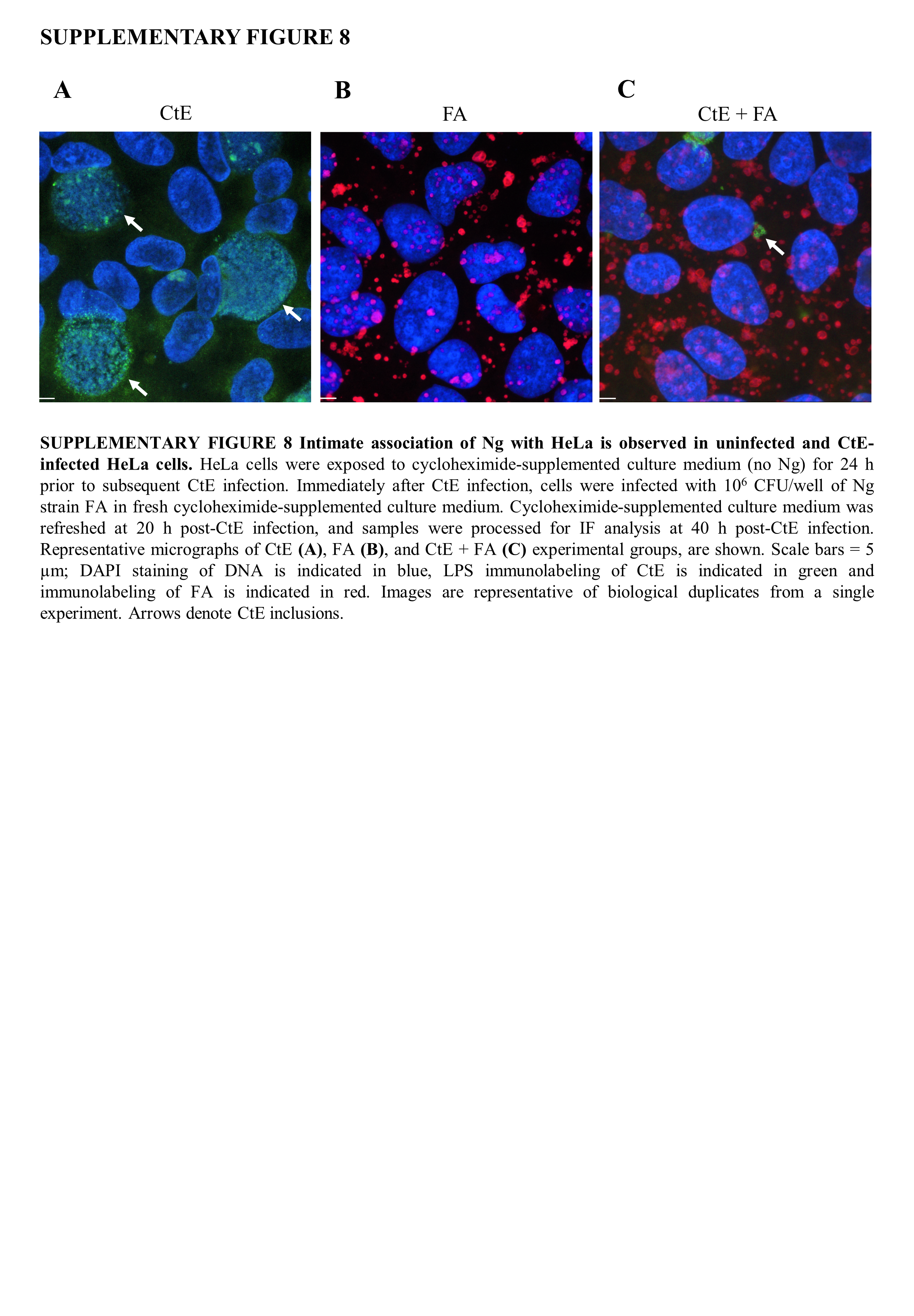

Supplement: Supplementary file 10 [file Image_8.tif]

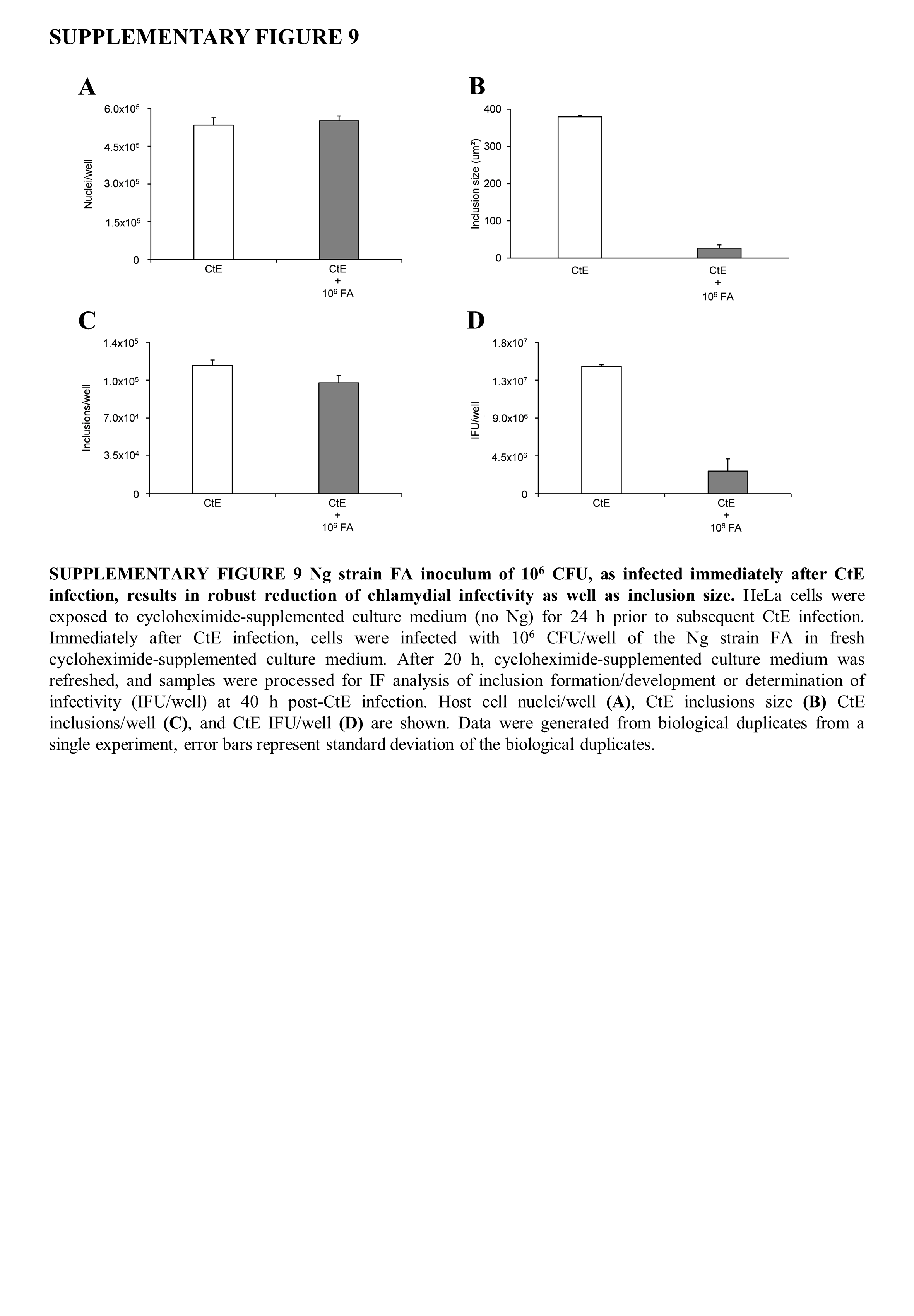

Supplement: Supplementary file 11 [file Image_9.tif]

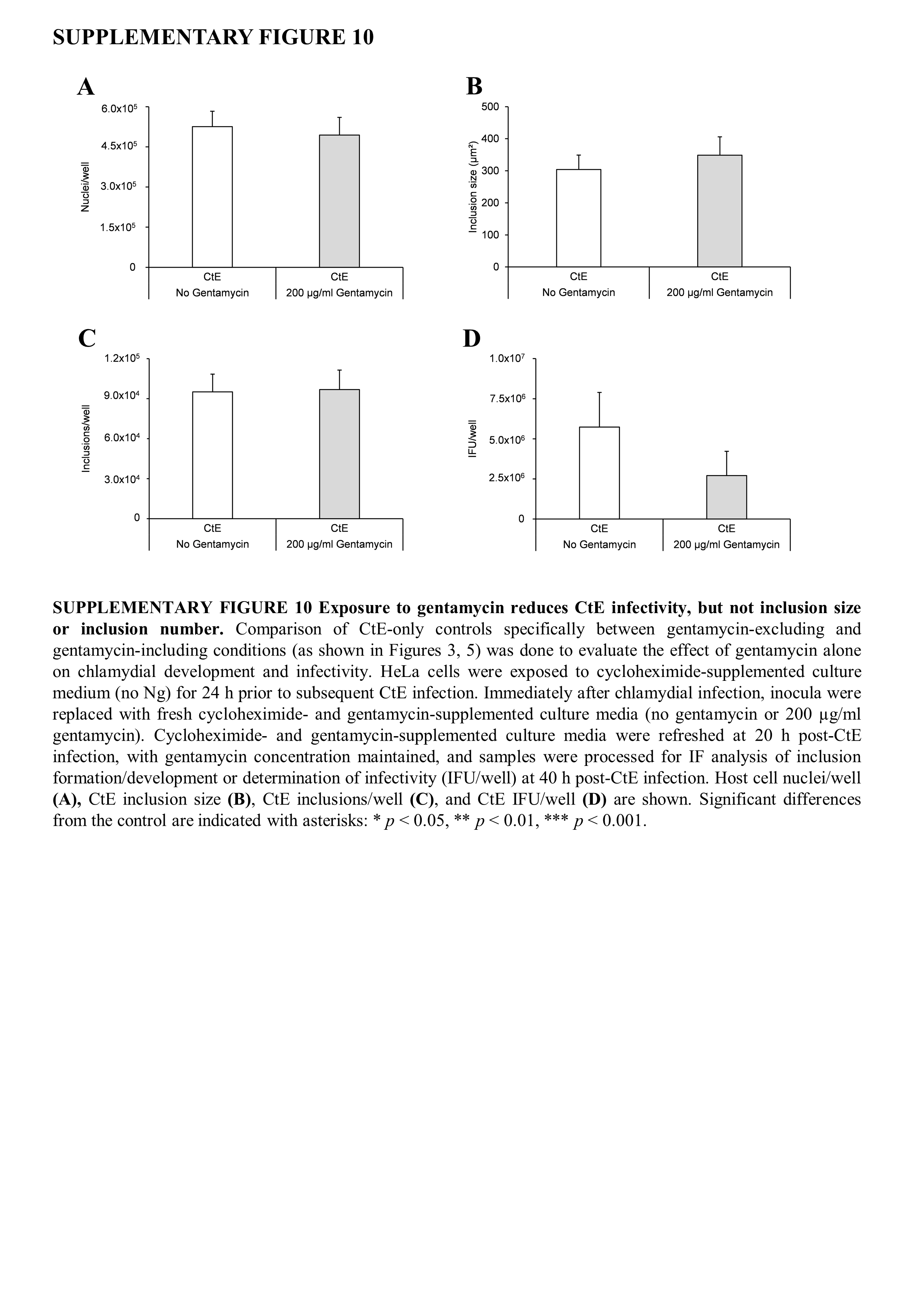

Supplement: Supplementary file 12 [file Image_10.tif]

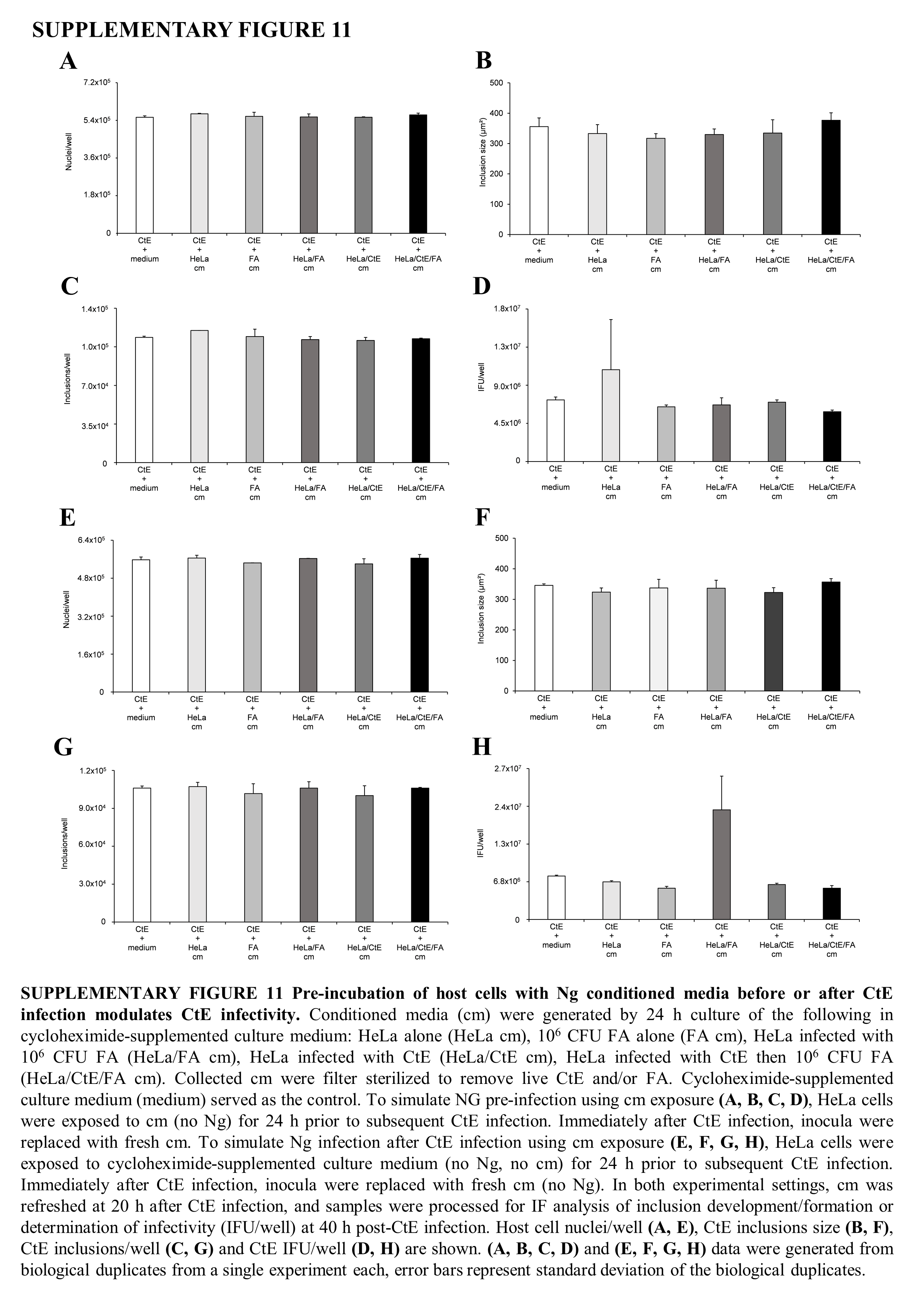

Supplement: Supplementary file 13 [file Image_11.tif]
