# Supplementary material for: Neisseria gonorrhoeae Limits Chlamydia trachomatis Inclusion Development and Infectivity in a Novel In Vitro Co-Infection Model
Source: Front Cell Infect Microbiol. 2022 Jul 7;12:911818. doi: 10.3389/fcimb.2022.911818 (PMC9300984; doi:10.3389/fcimb.2022.911818)
Supplement: Supplementary file 2 [file DataSheet_2.pdf]

**Supplementary Text 2_Media Composition**

| | | DMEM/F12 | | MEM NEAA (100X) | | | GlutaMAX (100X) | | | MEM AA (50X) | | |
|---|---|---|---|---|---|---|---|---|---|---|---|---|
| **COMPONENTS** | MW | mg/L | mM | mg/L | mM (100 X) | mM (1X) | mg/L | mM (100X) | mM (1X) | mg/L | mM (50 X) | mM (1X) |
| **AA** | | | | | | | | | | | | |
| L-Arginine hydrochloride | 211,00 | 147,35 | 0,70 | | | | | | | 6320,00 | 29,95 | 0,60 |
| L-Cystine | 240,00 | | | | | | | | | 1200,00 | 5,00 | 0,10 |
| L-Cystine hydrochloride-H2O | 176,00 | 17,65 | 0,10 | | | | | | | | | |
| L-Cystine 2HCl | 313,00 | 31,29 | 0,10 | | | | | | | | | |
| L- Histidine hydrochloride-H2O | 210,00 | 31,48 | 0,15 | | | | | | | 2100,00 | 10,00 | 0,20 |
| L-Isoleucine | 131,00 | 54,37 | 0,42 | | | | | | | 2620,00 | 20,00 | 0,40 |
| L-Leucine | 131,00 | 58,96 | 0,45 | | | | | | | 2620,00 | 20,00 | 0,40 |
| L-Lysine hydrochloride | 183,00 | 91,37 | 0,50 | | | | | | | 3625,00 | 19,81 | 0,40 |
| L-Methionine | 149,00 | 17,24 | 0,12 | | | | | | | 755,00 | 5,07 | 0,10 |
| L-Phenylalanine | 165,00 | 35,48 | 0,22 | | | | | | | 1650,00 | 10,00 | 0,20 |
| L-Threonine | 119,00 | 53,56 | 0,45 | | | | | | | 2380,00 | 20,00 | 0,40 |
| L-Tryptophan | 204,00 | | | | | | | | | 510,00 | 2,50 | 0,05 |
| L-Tyrosine | 181,00 | | | | | | | | | 1800,00 | 9,94 | 0,20 |
| L-Tyrosine disodium salt dihydrate | 261,00 | 55,81 | 0,21 | | | | | | | | | |
| L-Valine | 117,00 | 52,66 | 0,45 | | | | | | | 2340,00 | 20,00 | 0,40 |
| Glycine | 75,00 | 18,76 | 0,25 | 750,00 | 10,00 | 0,10 | | | | | | |
| L-Alanine | 89,00 | 4,46 | 0,05 | 890,00 | 10,00 | 0,10 | | | | | | |
| L-Asparagine | 132,00 | | | 1320,00 | 10,00 | 0,10 | | | | | | |
| L-Asparagine-H2O | 150,00 | 8,53 | 0,06 | | | | | | | | | |
| L-Aspartic acid | 133,00 | 6,66 | 0,05 | 1330,00 | 10,00 | 0,10 | | | | | | |
| L-Glutamic acid | 147,00 | 7,36 | 0,05 | 1470,00 | 10,00 | 0,10 | | | | | | |
| L-Gutamine | 146,00 | 365,10 | 2,50 | | | | | | | | | |
| L-Alanyl-L-Glutamine | 217,00 | | | | | | 43,4 | 200 | 2 | | | |
| L-Proline | 115,00 | 17,27 | 0,15 | 150,00 | 10,00 | 0,10 | | | | | | |
| L-Serine | 105,00 | 26,26 | 0,25 | 1050,00 | 10,00 | 0,10 | | | | | | |
| **Vitamins** | | | | | | | | | | | | |
| Biotin | 244,00 | 0,00 | 0,00 | | | | | | | | | |
| Choline chloride | 140,00 | 8,98 | 0,06 | | | | | | | | | |
| D-Calcium pantothenate | 477,00 | 2,12 | 0,00 | | | | | | | | | |
| Folic acid | 441,00 | 2,66 | 0,01 | | | | | | | | | |
| Niacinamide | 122,00 | | | | | | | | | | | |
| Pyridoxal hydrochloride | 203,62 | 2,00 | 0,01 | | | | | | | | | |
| Pyridoxine hydrochloride | 206,00 | 0,03 | 0,00 | | | | | | | | | |
| Ribloflavin | 376,00 | 0,22 | 0,00 | | | | | | | | | |
| Thiamine hydrochloride | 337,00 | 2,17 | 0,01 | | | | | | | | | |
| Vitamin B12 | 1355,00 | 0,68 | 0,00 | | | | | | | | | |
| i_(or myo)_Inositol | 180,00 | 0,01 | 0,00 | | | | | | | | | |
| **Inorganic Salts** | | | | | | | | | | | | |
| Calcium Chloride:H2O | 129,00 | 154,52 | 1,20 | | | | | | | | | |
| Cupric sulfate (CuSO4-5H2O) | 250,00 | 0,00 | 0,00 | | | | | | | | | |
| Ferric Nitrate (Fe(NO3)3"9H2O) | 404,00 | 0,05 | 0,00 | | | | | | | | | |
| Ferric sulfate (FeSO4-7H2O) | 278,00 | 0,42 | 0,00 | | | | | | | | | |
| Magnesium Chloride (anhydrous) | 95,00 | 28,57 | 0,30 | | | | | | | | | |
| Magnesium Sulfate (MgSO4) (anhyd.) | 120,00 | 48,83 | 0,41 | | | | | | | | | |
| Potassium Chloride (KCl) | 75,00 | 311,83 | 4,16 | | | | | | | | | |
| Sodium Chloride (NaCl) | 58,00 | 6999,50 | 120,68 | | | | | | | | | |
| Sodium Phosphate dibasic (Na2HPO4) anhydrous | 142,00 | 71,06 | 0,50 | | | | | | | | | |
| Sodium Phosphate monobasic (NaH2PO4-H2O) | 138,00 | 54,35 | 0,39 | | | | | | | | | |
| Zinc sulfate (ZnSO4-7H2O) | 288,00 | 0,43 | 0,00 | | | | | | | | | |
| **Other Components** | | | | | | | | | | | | |
| D-Glucose (Dextrose) | 180,00 | 3150,00 | 17,50 | | | | | | | | | |
| Hypoxanthine Na | 159,00 | 2,10 | 0,01 | | | | | | | | | |
| Linoleic Acid | 280,00 | 0,04 | 0,00 | | | | | | | | | |
| Lipoic Acid | 206,00 | 0,11 | 0,00 | | | | | | | | | |
| Phenol Red | 376,40 | 8,31 | 0,02 | | | | | | | | | |
| Putrescine 2HCl | 161,00 | 0,08 | 0,00 | | | | | | | | | |
| Sodium Pyruvate | 110,00 | 55,05 | 0,50 | | | | | | | | | |
| Thymidine | 242,00 | 0,37 | 0,00 | | | | | | | | | |
